# In-depth assembly of organ and development dissected *Picrorhiza kurroa* proteome map using mass spectrometry

**DOI:** 10.1186/s12870-021-03394-8

**Published:** 2021-12-22

**Authors:** Manglesh Kumari, Upendra Kumar Pradhan, Robin Joshi, Ashwani Punia, Ravi Shankar, Rajiv Kumar

**Affiliations:** 1 Biotechnology Division, CSIR-Institute of Himalayan Bioresource Technology (IHBT), Palampur, 176061 HP India; 2 Academy of Scientific and Innovative Research (AcSIR), Ghaziabad, 201002 India; 3 Studio of Computational Biology & Bioinformatics (Biotech Division), The Himalayan Centre for High-throughput Computational Biology (HiCHiCoB, A BIC Supported by DBT, India), CSIR-IHBT, Palampur, HP, 176061, India; 4 Present address: ICAR-Indian Agricultural Statistics Research Institute, Library Avenue, Pusa, New Delhi, Delhi 110012 India

**Keywords:** *Picrorhiza kurroa*, Proteome, PTMs, RBPs, Picrosides, Metabolites

## Abstract

**Background:**

*Picrorhiza kurroa* Royle ex Benth. being a rich source of phytochemicals, is a promising high altitude medicinal herb of Himalaya. The medicinal potential is attributed to picrosides i.e. iridoid glycosides, which synthesized in organ-specific manner through highly complex pathways. Here, we present a large-scale proteome reference map of *P. kurroa,* consisting of four morphologically differentiated organs and two developmental stages.

**Results:**

We were able to identify 5186 protein accessions (FDR < 1%) providing a deep coverage of protein abundance array, spanning around six orders of magnitude. Most of the identified proteins are associated with metabolic processes, response to abiotic stimuli and cellular processes. Organ specific sub-proteomes highlights organ specialized functions that would offer insights to explore tissue profile for specific protein classes. With reference to *P. kurroa* development, vegetative phase is enriched with growth related processes, however generative phase harvests more energy in secondary metabolic pathways. Furthermore, stress-responsive proteins, RNA binding proteins (RBPs) and post-translational modifications (PTMs), particularly phosphorylation and ADP-ribosylation play an important role in *P. kurroa* adaptation to alpine environment. The proteins involved in the synthesis of secondary metabolites are well represented in *P. kurroa* proteome. The phytochemical analysis revealed that marker compounds were highly accumulated in rhizome and overall, during the late stage of development.

**Conclusions:**

This report represents first extensive proteomic description of organ and developmental dissected *P. kurroa*, providing a platform for future studies related to stress tolerance and medical applications.

**Supplementary Information:**

The online version contains supplementary material available at 10.1186/s12870-021-03394-8.

## Background

The Himalaya is an important repository of medicinal and aromatic plants that are a rich source of novel compounds for pharmaceutical industry. Most of them are confined to high-altitude regions that are characterized by extremes temperatures, radiations, drought, snow cover and partial pressure of gases. These plant species synthesize extensive arrays of secondary metabolites having medicinal importance. Owing to the successful acclimatization in extreme environments, high-altitude plants can serve as a model for elucidation of combinatorial stress tolerance mechanisms. *Picrorhiza kurroa* Royle ex Benth. (Family Plantaginaceae), a perennial herb of Himalaya (3000–5000 masl), has been used in traditional and modern medicine systems as hepatoprotective, antiperiodic, cholagouge, stomachic, antiamoebic, antioxidant, antihelmintic, antiinflammatory, cardiotonic, laxative, carminative and expectorant [1–3]. The medicinal potential is mostly ascribed to iridoid glycosides i.e. picrosides, which are used in herbal formulation of more than 2000 drugs [1, 4]. Morphologically, plants are herbaceous with an elongated rhizome (rootstock) and aerial portion consists of basal leaves and terminal inflorescence (flowering spike) [5]. *P. kurroa* propagates vegetatively as young buds on rhizomes, which subsequently develops as a new rhizome with autonomous root and shoot sections [6]. In addition, rhizome survives underground during snow cover whereas, shoot emerges rapidly during onset of suitable growth conditions. *P. kurroa* synthesizes active phytochemicals in tissue/organ-specific manner through highly complex biosynthetic pathways [7]. Due to active phytochemical ingredients, the global annual demand of this plant is increased to 500 tons, whereas its supply is only 375 tons [1, 8]. Consequently, proteomic technologies provide one of the best choices for the functional analysis of translated parts of the genome. In general, plant proteome studies have increased over the past few years, only the model organisms and food crops continue to be studied. Since, proteins can be better conserved, the identification of non-model protein accessions by comparing them to familiar orthologous proteins would result into functional identification across different species [9]. This cross species identification of proteins is the best approach for extensive study of gene products, wherever genome is uncharacterized [10]. The establishment of species-specific proteome maps using multidimensional fractionations of various cell, tissue or organ types has been essential for better understanding of underlying molecular basis of the plant system.

The proteome reference map has been established for a variety of cells, tissues, organs and organelles from non-model plant species such as *Miscanthus sinensis*, *Pisum sativum, Gingo biloba*, *Triticum aestivum*, *Arachis hypogaea*, *Quercus ilex*, *Pinus radiata*, *Tectona grandis* to model plants like *Arabidopsis thaliana*, *Oryza sativa*, *Glycine max*, *Solanum lycopersicum*, *Medicago truncatula* and *Zea mays* based upon the two-dimensional gel electrophoresis (2D-GE) due to its better resolving power [11–25]. Recently, alternative approaches such as gel-free techniques have also been utilized for the construction of proteome map in *Triticum aestivum* and *Arabidopsis thaliana* [26, 27]. However, bottom-up, gel-free approaches are reported less-efficient in non-model systems due to loosing connectivity between protein-derived peptides [28]. Furthermore, taking advantage of one-dimensional gel electrophoresis (1D-GE) coupled with liquid chromatography (LC) prior to mass spectrometry (MS), a high density *Arabidopsis* proteome was assembled [29]. The key benefit of this combinatorial approach is to facilitate the use of SDS in 1D-GE that ensures solubility of proteins during size separation and reduction in sample complexity before LC, which increases the probability of identifying low abundant proteins [30]. Previous reports have also compared 1D-GE to other pre-fractionation approaches such as isoelectric focusing, strong cation exchange chromatography and protein reversed chromatography at peptide and protein level, which revealed that 1D-GE is most effective pre-fractionation method for greater  proteome coverage [31, 32].

Here, we provided 1D-GE-nanoLC-MS/MS based proteomic datasets, which collectively assemble first proteome map of *P. kurroa* covering all four major organs and two developmental stages. Together or separately, these organs function in response to chronic environmental fluctuations at high altitudes. This study has entitled the dissection of developmental and organ-specific proteomes, pathway-centric comparison with functional differences as well as uncovered the protein/enzyme basis of specialized metabolites accumulation in different organs. Further, the identification of stress-responsive proteins, various PTMs and RBPs would provide insights into stress tolerance potential of *P. kurroa*.

## Results

### Overview of organ and developmental dissection of *P. kurroa* proteome

**Fig. 1:**
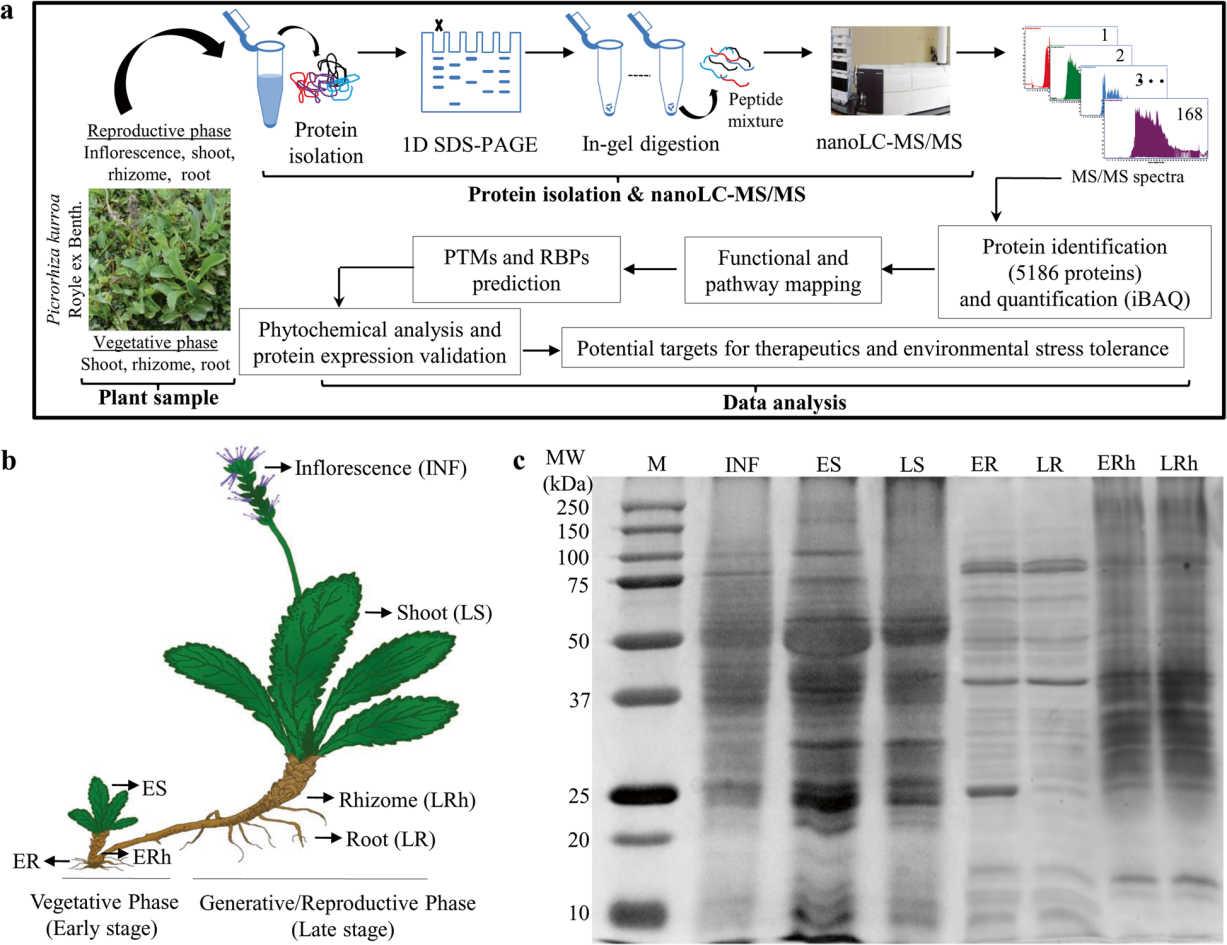
A brief summary of proteome analysis in *P. kurroa*. **A** An illustration of the entire workflow employed for *P. kurroa* whole proteome. **B** Schematic representation of organ and developmental analyzed samples. The abbreviations used are INF, Inflorescence; LS, Late shoot; LRh, Late rhizome; LR, Late root; ES, Early shoot; ERh, Early rhizome; ER, Early root. **C** Representative SDS-PAGE electropherogram. Approximately, 25 µg of proteins from four organs at key developmental stages were fractionated using 12.5% SDS-PAGE, stained with CBB and used abbreviations are defined in B

An illustration of the entire workflow was represented in Fig. 1A. Root, shoot and rhizome were harvested from both early (vegetative/before flowering) and late (reproductive/after flowering) stage, whereas inflorescence was collected from various plants at late stage, which represent seven samples (Fig. 1B). To reduce the impact of biological variation, each of these samples were pool of at least eight different plants. To fractionate proteins, SDS-PAGE from seven different samples of *P. kurroa* was performed*.* The signal pattern observed in Coomassie brilliant blue (CBB) stained 1D gel was found to be dissimilar for all major organs (Fig. 1C). Although conventional Coomassie staining is less sensitive as compared to silver staining, but quite more simple, suitable and quantitative for downstream applications. Each sample lane was split into eight fractions, which were trypsinized and subsequently analyzed using nanoLC coupled with Agilent 6560 Q-TOF mass spectrometer. The resulted tandem mass spectra were searched against NCBI protein database (2018) containing predicted proteins of all green plants and generated a total of 5186 non-redundant protein accessions (FDR < 1%, peptide score ≥ 3 and SPI (%) ≥ 60) (Table S1). The proteins having at least one distinct peptide and clustering of similar protein based on sequence homology into same group were considered as positive identification [33].

**Fig. 2:**
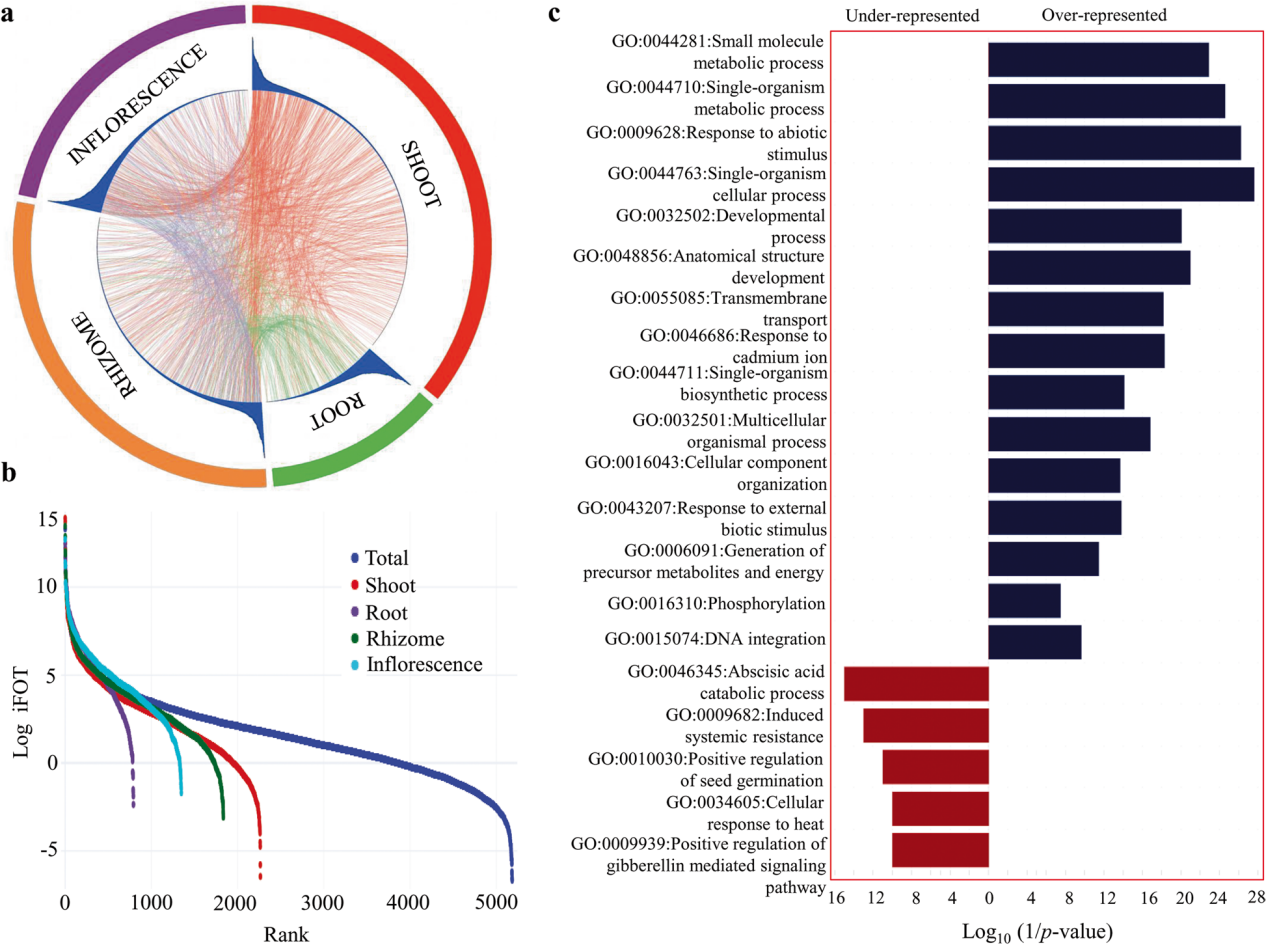
Comprehensive map of *P. kurroa* proteome. **A** A circular proteome map showing similarities and dissimilarities among four organs. The relative abundance (iFOT) of each protein within an organ is represented with a blue histogram. **B** The dynamic ranges of *P. kurroa* proteome measured by protein abundance on logarithm scale (log_2_ iFOT). **C** GO categories enrichment analysis of *P. kurroa* from the aspect “biological process” where over-represented and under-represented terms are shown in blue and red color, respectively, when compared with a customized reference of green plant species viz*. Z. mays*, *G. max*, *A. thaliana*, *S. tuberosum*, *T. aestivum*, *N. tabacum*, *P. sativum*, *O. sativa* and *H. vulgare* (Fisher’s exact test and Bonferroni adjustment; *p* < 0.05)

A summary of matched spectra, distinct peptides and proteins identified are displayed in Table 1. On average, the whole proteome ranging from a minimum of 793 protein accessions in root to a maximum of 2267 protein accessions in shoot indicates *P. kurroa* shoot possesses the highest, while root having lowest protein content (Table 1; Table S1). These results are in agreement with our previous report that suggests the pattern of total protein accumulation is organ-specific; shoot/leaf have highest, while root have lowest crude protein content in *P. kurroa* [7]. We provided a circular proteome map of protein abundance in *P. kurroa* to describe the similarities and differences among organ sub-proteomes (Fig. 2A). This displayed protein groups overlapped among diverse organs in addition to explicitly found in only one organ. Next, the depth of our *P. kurroa* proteome was assessed by a critical figure of dynamic range (Fig. 2B). The whole proteome dynamic range spanned around six orders of magnitude, when measured based upon protein abundance (iFOT). Similar dynamic ranges of protein expression have also been reported in higher eukaryotes like yeast and *Populus* that were directed towards deep proteome coverage [34, 35].

**Table 1:** An outline of the number of matched spectra, distinct peptides and identified proteins through 1D-GE-nanoLC-MS/MS in *P. kurroa*. Different alphabets represent statistically significant values (*p* < 0.05) as determined by one-way ANOVA followed by Tukey’s HSD post-hoc test for each parameter individually in R v. 4.0.0

Plant organ	Spectra	Distinct Peptides	Proteins
Inflorescence	8107^b^	1495^c^	1348^c^
Shoot	10749^a^	2531^a^	2267^a^
Early shoot	4837^de^	1184^d^	1097^d^
Late shoot	8242^b^	1596^c^	1433^c^
Rhizome	10536^a^	2070^b^	1837^b^
Early rhizome	5095^d^	1081^d^	970^de^
Late rhizome	6990^c^	1199^d^	1081^d^
Root	4920^de^	841^e^	793^ef^
Early Root	4385^e^	678^ef^	642^f^
Late Root	3175^f^	490^f^	396^ g^
**Total**	**25,069**	**5922**	**5186**

 The raw files were searched against green plant database (NCBInr). The identified proteins (5186) comprise from nine plants species viz*. Zea mays* (27.76%), *Glycine max* (26.41%), *Arabidopsis thaliana* (21.63%), *Solanum tuberosum* (17.62%), *Triticum aestivum* (3.97%), *Nicotiana tabacum* (1.13%), *Pisum sativum* (0.67%), *Oryza sativa* (0.56%) and *Hordeum vulgare* (0.21%). Therefore, we performed gene ontology (GO) enrichment of total proteins compared with a customized reference database of aforementioned nine plant species using Fisher’s exact test to determine the significance of over or under-represented GO terms. This analysis revealed a total of 359 enriched GO terms that were classified into cellular component, molecular function and biological process (Table S3). The five topmost enriched biological process/GO categories include single-organism cellular process, response to abiotic stimulus, single-organism metabolic process, small molecule metabolic process and anatomical structure development (Fig. 2C). However, the under-represented biological process/GO categories include proteins involved in abscisic acid catabolic process, induced systemic resistance, positive regulation of seed germination and gibberellic acid mediated signaling pathway (Fig. 2C). The most enriched molecular functions comprise purine ribonucleoside triphosphate binding, ATP binding, hydrolase activity, while cell periphery, cytoplasm and chloroplast were found to be highly enriched cellular components (Table S3).

### Comprehensive map of *P. kurroa* organ proteomes

**Fig. 3:**
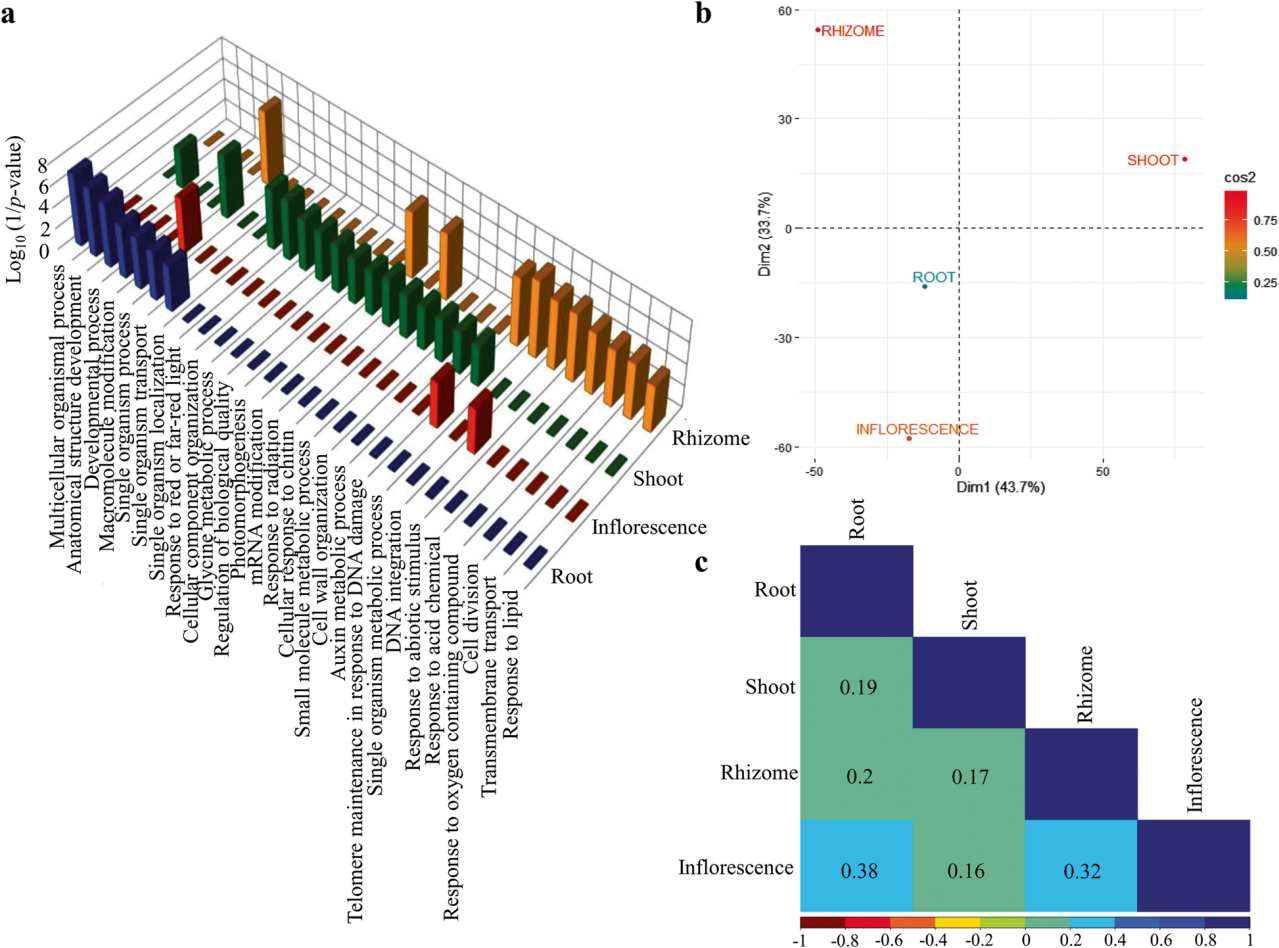
Spatial diversity of *P. kurroa* proteome. **A** The over-represented GO categories of proteins from the aspect “biological process” in each of the organ as compared to the entire identified proteins of *P. kurroa* (Fisher’s exact test and Bonferroni adjustment; *p* < 0.05). **B** Principal component analysis of *P. kurroa* organs based upon the protein abundance profile. **C** The correlation matrix among four *P. kurroa* organs representing Pearson correlation coefficients (r)

A functional view of *P. kurroa* sub-proteomes was generated to compare the GO category enrichment of each organ (Fig. 3A; Table S3). Most of the enriched GO terms were organ-specific, whereas single-organism process was over-represented in all organs. With reference to specific organs, single-organism metabolic process and response to abiotic stimulus were highly enriched in inflorescence. In rhizome, proteins were over-represented for small molecule metabolic process, DNA integration, response to abiotic stimulus, response to oxygen-containing compounds, cell division and transmembrane transport. The most abundant categories in shoot include response to red/far-red light, cellular component organization, glycine metabolic process, regulation of biological quality, photomorphogenesis and mRNA modification, beside others. In contrast, proteins were enriched for developmental process, multicellular organismal process, anatomical structure development, macromolecule modification, transport and localization in root. Thus, each *P. kurroa* organ can be assigned a specific proteome, which reflects organ specialized functions. Further, we exemplify this using principal component analysis (PCA) based on protein abundance in each organ. The first two principal components (Dim 1 and Dim 2) explained 77.4% of variation and their plotting allocate the clear separation of all organs (Fig. 3B). Similarly, the pairwise correlation value of organ proteomes ranges from a minimum of 0.16 between shoot and inflorescence to a maximum of 0.38 between root and inflorescence (Fig. 3C). The weak correlation coefficients among *P. kurroa* organs indicate the organ specialization, which is reflected by specific types of protein abundance.

**Fig. 4:**
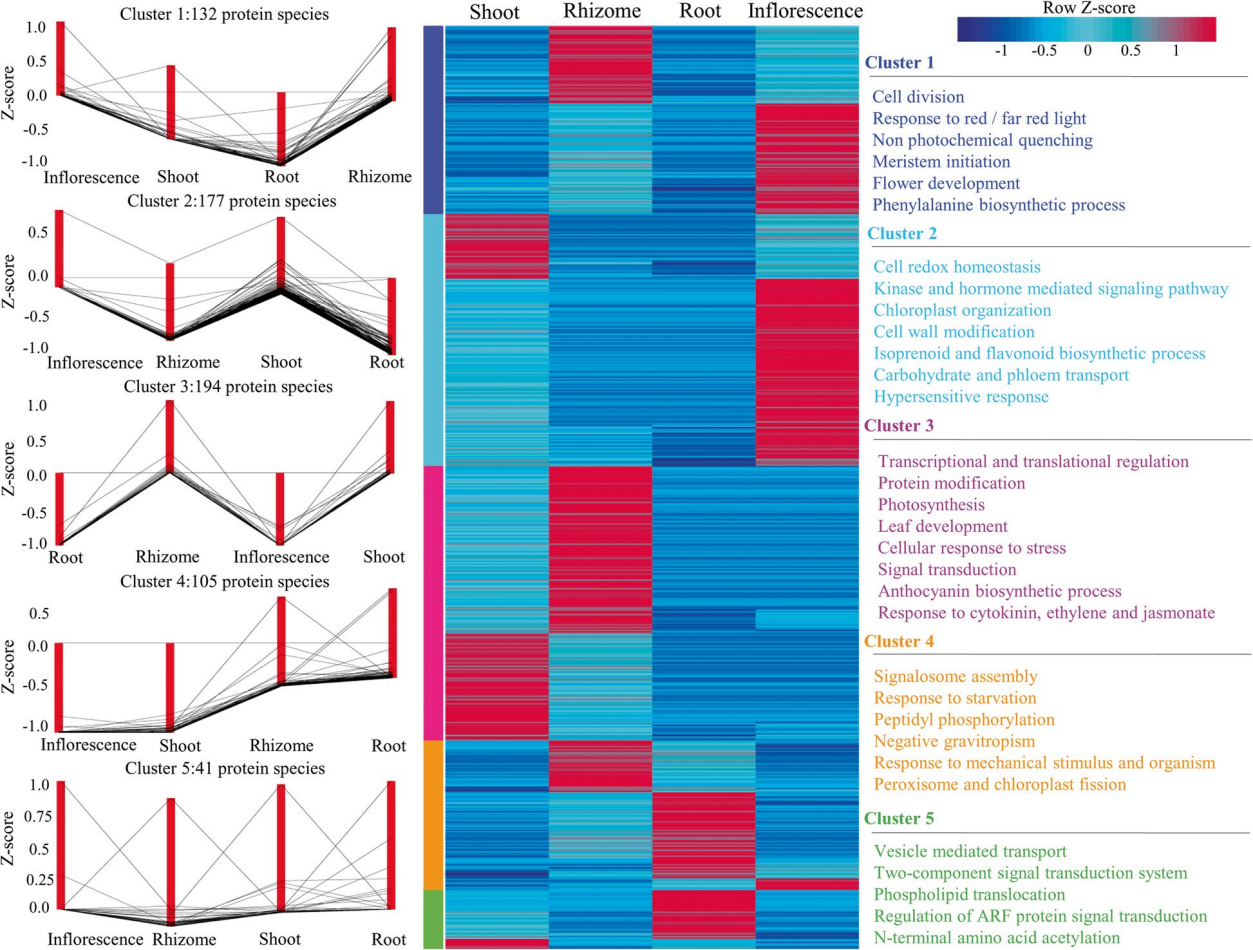
Five protein clusters revealed by hierarchical clustering analysis (silhouette index > 0.66). Left panel: The co-expression pattern of proteins in representative five clusters; Centre panel: The heat map of 650 protein accessions was analyzed based on protein abundance (z-scores); Right panel: The representative GO terms enrichment from the aspect “biological process” of each cluster

Hierarchical cluster analysis was applied to 650 protein accessions (those shared by organs), which resulted in five protein clusters suggests functions of *P. kurroa* organs are distinct (Fig. 4). Across the observed five clusters, the number of protein species ranged from 41 in cluster 5 to 194 in cluster 3 (Table S4). Cluster 1 seemed to be associated with protein abundance in inflorescence and rhizome, whereas cluster 2 comprised of more abundant proteins in aerial portion of *P. kurroa* i.e. inflorescence and shoot. Cluster 3, the largest one connected with predominant proteins in shoot and rhizome, which showed characteristic biological organization. The protein groups belonging to cluster 4 are found to be most abundant in root and fairly in rhizome, however the smallest cluster 5 showed protein abundance in root and a little bit in shoot.

### Pathway analysis between early and late developmental stages

**Fig. 5:**
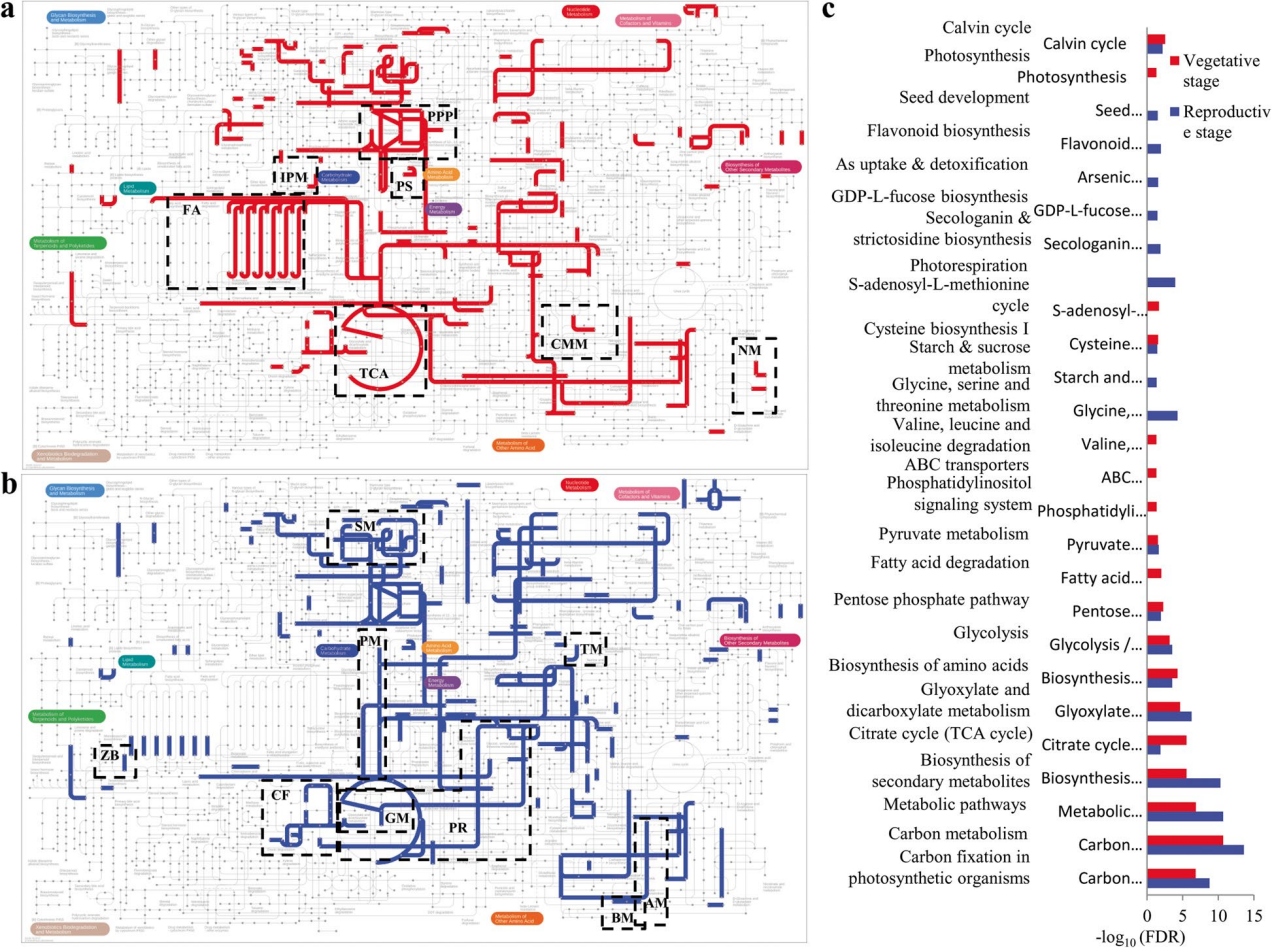
Pathway-centric comparison of *P. kurroa* developmental stages. Early and late stages protein groups with assigned functions (Enzyme EC number) were mapped to KEGG pathways using iPath v. 3.0. **A** Vegetative/early stage and **B** Reproductive/late stage. Highlighted pathways (dashed boxes) include photosynthesis (PS), tricarboxylic acid cycle (TCA), pentose phosphate pathway (PPP), fatty acid degradation (FA), inositol phosphate metabolism (IPM), cysteine and methionine metabolism (CMM), nicotinamide metabolism (NM), starch and sucrose metabolism (SM), carbon fixation (CF), pyruvate metabolism (PM), photorespiration (PR), glyoxylate and dicarboxylate metabolism (GM), zeatin biosynthesis (ZB), biotin metabolism (BM), β-alanine metabolism (AM), taurine, and hypotaurine metabolism (TM). (C) KEGG-pathway enrichment of protein groups (Fisher’s exact test; *p* < 0.05) in vegetative and reproductive stages as compared to a customized reference of green plant species viz*. Z. mays*, *G. max*, *A. thaliana*, *S. tuberosum*, *T. aestivum*, *N. tabacum*, *P. sativum*, *O. sativa,* and *H. vulgare*

To visualize the metabolic differences between early and late stages, protein groups having assigned functions (Enzyme EC number) were mapped into KEGG derived metabolic pathways using iPATH (v.3.0; Fig. 5A-B). In addition, pathway enrichment in comparison with aforementioned nine plant species could provide a functional view and thus, expand the biological information regarding *P. kurroa* developmental stages (Fig. 5C). Vegetative phase was found to be significantly enriched for photosynthesis, Calvin cycle, biosynthesis of amino acids, TCA cycle, pentose phosphate pathway, fatty acid degradation, phosphatidylinositol signaling, ABC transporters, Cys biosynthesis, Val, Leu, Ile degradation and S-adenosyl-L-methionine cycle. Relative to the vegetative phase, diverse metabolic pathways such as carbon metabolism, glyoxylate and dicarboxylate metabolism, starch and sucrose metabolism, Gly, Ser and Thr metabolism were found to be enriched in reproductive phase. In addition, processes involved in the production of secondary metabolites like secologanin, strictosidine, GDP-L-fucose and flavonoids besides seed development were significantly enriched in the reproductive phase. Furthermore, proteins linked with biotin metabolism, β-alanine metabolism, zeatin biosynthesis, taurine and hypotaurine metabolism are exclusively observed in the reproductive stage of *P. kurroa*.

### Proteome-wide prediction of RNA binding proteins (RBPs) and post-translational modifications (PTMs)

The RBPs repertoire responds to a magnitude of biological and environmental cues. The plants RBPs have been less studied compared to other organisms, however studies on *A. thaliana* and *O. sativa* showed that many of the RBPs are unique to plants, which suggested their plant-specific functions [36]. In the recent past, it has been well established that besides their role in various developmental processes, they are also essential for plant adaptation to various unfavorable environmental conditions [37]. To identify putative RBPs in *P. kurroa* proteome, domain analysis was performed for proteins that harbor classical, non-classical or unknown RNA-binding domains (RBDs) reported so far in plants [38]. A total of 1272 putative *P. kurroa* RBPs with 586 RBDs were identified that represents 24.5% of the whole proteome (Table S5). Most of the putative *P. kurroa* RBPs comprised of protein kinase (Pkinase), leucine-rich repeat (LRR_8), LRR N-terminal (LRRNT_2), pentatricopeptide repeat (PPR; PPR_2), tyrosine kinase (Pkinase_Tyr), nucleotide-binding (NB-ARC), cytochrome P450 (p450) and ABC transporter (ABC_tran) domains, which reflect their key role in gene regulation, phosphorylation, protein–protein interaction, synthesis of secondary metabolites, transmembrane translocator and molecular switch for cycling between ADP and ATP bound forms.

**Fig. 6:**
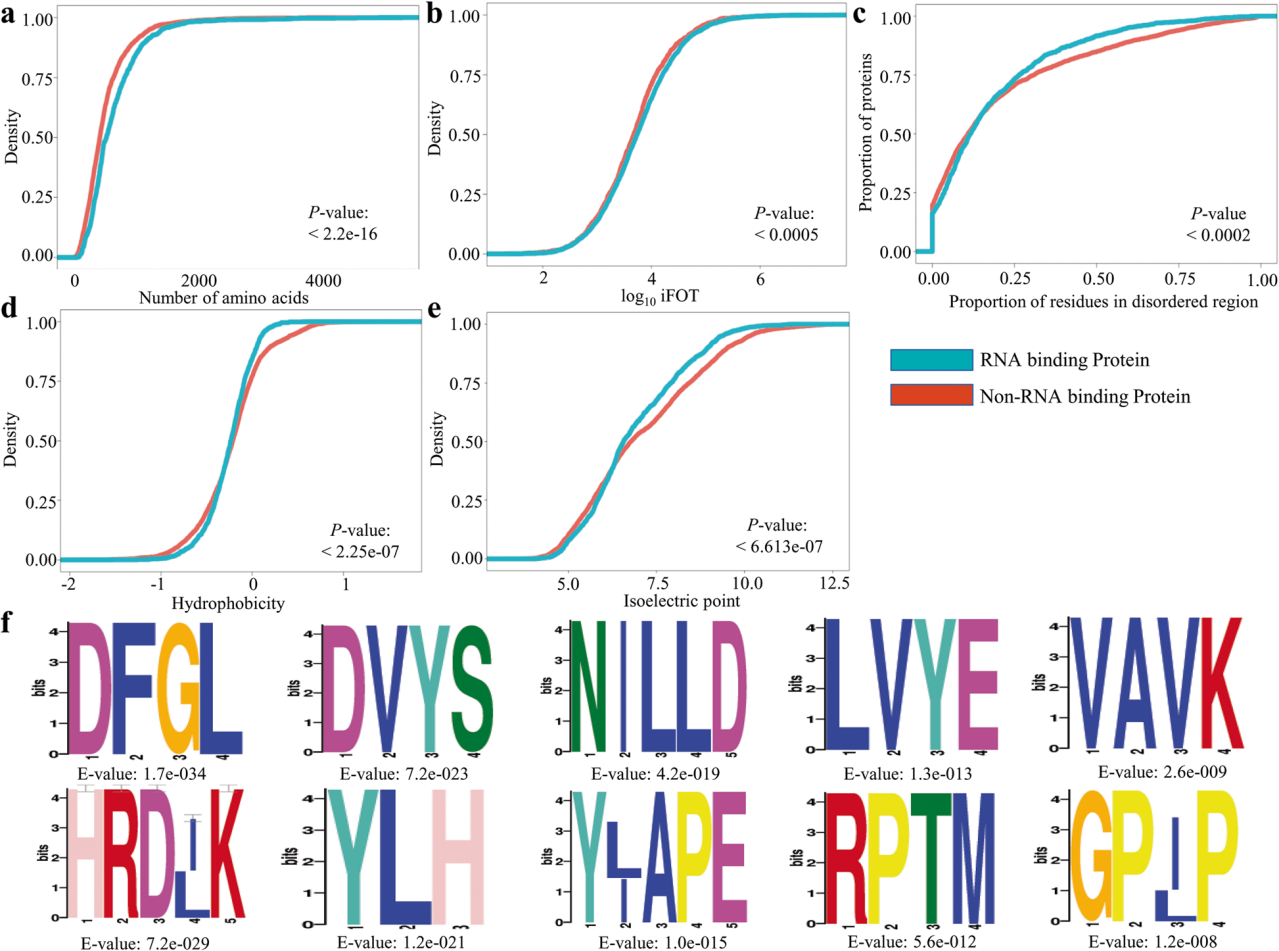
Biophysical characteristics and sequence features of *P. kurroa* putative RBPs. **A** Density of protein length, **B** Density of log_10_ iFOT, **C** Proportion of residues in disorder region, **D** Hydrophobicity, and **E** Isoelectric point. Significant differences among putative RBPs and non-RBPs were analyzed using Kolmogorov–Smirnov test (*p* < 0.01). **F** The top ten most enriched amino acid motifs in *P. kurroa* putative RBPs analyzed by DREME software (E-value < 0.05)

Next, we validate *P. kurroa* putative RBPs for their characteristic features by analyzing the physicochemical properties of RBPs in comparison with non-RBPs. Both putative RBP and non-RBP groups span the full array of protein length and abundance with some propensity in the direction of larger length and higher abundance for RBPs compared with non-RBPs (Fig. 6A-B) reflecting similar observations as reported previously [38]. Moreover, putative RBPs significantly shifted towards an increased proportion of residues in disordered regions and lower hydrophobicity (Fig. 6C-D) as reported earlier in *Arabidopsis* [38]. Thus, *P. kurroa* putative RBPs displayed the biophysical properties anticipated of bonafide RBPs except for the isoelectric point (Fig. 6E). In accordance, we also found thirty-six enriched amino acid motifs of putative RBPs generally comprised of disordered sequences (Fig. 6F; Table S5). Most of these conserved sequence motifs belong to proteins/enzymes/domains that harbor kinase or phosphatase activity, intracellular trafficking, localization signals, ubiquitin conjugation, protein–protein interaction with regulatory proteins, nucleotide triphosphate binding and RNA helicase activity. Overall, *P. kurroa* putative RBPs also showed significant biases in physicochemical features.

In order to gain structural and functional information of proteins, PTMs were predicted for the total proteome data set using ModPred (Table S6). Notably, phosphorylation (S, T & Y) and ADP-ribosylation (R) were detected at approximately 1.5 and 1.2% of total PTMs at peptide level, respectively suggesting their key role in *P. kurroa* developmental and adaptation processes. Further, domain analysis also highlighted Pkinase to be the most enriched domain in whole proteome dataset (Table S5). In addition to the above mentioned PTMs, *P. kurroa* proteome also demonstrated a significant number of additional PTMs such as carboxylation, ubiquitination, methylation, acetylation and SUMOylation (Table S6).

## Discussion

In order to determine protein atlas of *P. kurroa*, morphologically differentiated four major organs at two developmental stages were chosen for 1D-GE that have the advantage of protein solubilization during fractionation prior to nanoLC coupled with MS for deeper proteome coverage. We used phenol-based protocol for efficient protein extraction that has potential to generate highly pure samples as other plant compounds partition into a discrete aqueous phase from protein-enriched phenol phase. The top ranked proteins detected in *P. kurroa* total proteome include rubisco large subunit (chloroplast) and ribulose bisphosphate carboxylase (Rubisco). Other most abundant, highly ranked proteins comprised serine carboxypeptidase 45-like, pentatricopeptide repeat-containing protein At1g69290-like, rubisco activase, uncharacterized LOC100305517, glutathione S-transferase tau 7 and ATP synthase CF1 beta subunit (Table S2). In agreement with the depleted GO terms in *P. kurroa* (Fig. 2C), seed dormancy is common in alpine plants to increase the adaptability towards unfavorable environment. Additionally, low seed fertility in rhizomatous plants developed for their asexual/vegetative reproduction. The plant hormones, predominantly gibberellin and abscisic acid are the foremost endogenous determinants of seed germination and dormancy by acting antagonistically; abscisic acid positively modulates the dormancy, whereas gibberellin enhances germination [39]. Moreover, under-representation of gibberellic acid dependent signaling pathways might be associated with picroside biosynthesis. A previous study reported that gibberellin downregulates the picroside biosynthesis as both follow the same biosynthetic routes i.e. mevalonate and non-mevalonate pathways [40]. Further, our initial observation suggests that 24.5% of *P. kurroa* proteome harbors putative RBPs that could be involved in plant development and adaptation to the alpine environment, however further experimental studies will be required.

It is well established that protein phosphorylation is an important PTMs that integrates external and internal stimuli, regulates carbon–nitrogen metabolism and redox signaling in plants [41]. As *P. kurroa* proteome was found to be extremely enriched for “response to abiotic stimulus” and “metabolic process”, this provide us a clue that external abiotic stimuli might affect cellular and metabolic processes through protein phosphorylation in *P. kurroa*. However, ADP-ribosylation in the plant system has received less attention compared with the animal system, it plays a significant role in plant response to diverse biotic and abiotic stresses through implication in cellular processes like DNA repair [42]. Since, ADP-ribosylation can be catalyzed by ADP-ribosyl transferase in vitro and in vivo, the identification of 89 ribosylated peptides from *P. kurroa* provides information about the substrate identification. Thus, we can hypothesize that PTMs particularly, phosphorylation and ADP-ribosylation beside others can play an important role in *P. kurroa* adaptation against environmental fluctuations prevailing at high altitude.

### Spatial profiling of* P. kurroa* organ proteomes reflect functional diversity

To generate proteome map of *P. kurroa*, sub-proteomes of the four major organs were integrated. Moreover, individual proteome at vegetative and reproductive stages of development was incorporated for each organ, which represents organ sub-proteomes. We postulate that relatively higher abundance and specificity of proteins in an organ contribute significant organ specialized functions. For example, proteins associated with flower and seed development, fruit setting, flavonoid biosynthesis, embryogenesis, cell expansion, plant defense towards chilling and oxidative stress were found to be relatively abundant in inflorescence, which include cell wall invertase, methionine synthase, SCY1-like protein, transparent testa 12, glutathione S-transferase tau 7, N-acetyltransferase HLS1-like, lysM domain receptor-like kinase 3, NAC transcription factor, pentatricopeptide repeat-containing protein, exostosin family protein, F-box/LRR-repeat protein 10-like and AP2/B3-like transcriptional factor family protein [43–46]. Proteins linked with cell division, cell–cell communication, carbon fixation, system development, ubiquitination, signaling, protein folding, metabolism and stress response were found to be relatively more abundant in shoot. A few of these include ribulosebisphosphate carboxylase, carbonic anhydrase, long-chain acyl-CoA synthetase, WPP domain associated protein, MADS-box protein AGL19, stromal 70 kDa heat shock related protein, glutathione transferase 23, taxadiene 5-alpha hydroxylase, laccase precursor, LRR receptor-like serine/threonine protein kinase, two-component response regulator-like APRR2, RuBisCO large subunit-binding protein subunit alpha, F-box/kelch-repeat protein, chaperonin-60 alpha and heat shock protein 70.

Protein groups that comparatively abundant in root have a prominent biological role in root elongation, transport, root hair formation, differentiation, cell division, carbohydrate metabolism and defense response. The most abundant protein groups in root are alpha tubulin; significant impact on root development [47], ARF guanine-nucleotide exchange factor GNOM-like; involved in membrane trafficking and cytoskeleton dynamics to drive tip growth in root hairs [48] and others such as arabinogalactan 21 and transducin / WD-40 repeat family protein. Various channels/transporters like amino acid permease 4 (amino acid transport across membrane), potassium channel AKT6 (modulate root hair K^+^ uptake), pleiotropic drug resistance protein 2-like (efflux antifungal or antibacterial metabolites), glutathione S-conjugate transporting ATPase (remove xenobiotics and endogenous toxicants out of cytosol) and ABC transporter G family member 11-like (metal transport and excretion of exudates) either enable the plant access for nutrients or impart defense response [49, 50]. Diverse signaling, cell cycle and defense/stress-responsive proteins abundant in root include peptidyl-prolyl cis–trans isomerase, receptor protein kinase, Phox (PX) domain, cucumisin-like, class III peroxidase, NAP1-like, MACPF domain-containing protein CAD1-like, HERK 1-like, phospholipid-transporting ATPase 9-like, LEA protein 2-like, SGT1-1, RPA 70 kDa subunit, CDPK 33 and serine/threonine kinase that indirectly affect root growth and development [51]. This suggests that high-altitude alpine environment advances the proliferation of root system to gain access for soil water and nutrients, which would be useful in developing strategies for greater soil exploration capacities.

At ecosystem level, rhizome is associated with soil protection and prevents erosion [52]. It is well known that rhizome stores energy reserves (starch, proteins) and allocates nutrients for regrowth. The abundance pattern revealed proteins associated with secondary metabolic pathways (2-oxoglutarate-dependent dioxygenase AOP1.2-like and 4-coumarate–CoA ligase-like 1), energy metabolism (alpha subunit of ATPase, aconitate hydratase 3, ATP synthase CF1 beta subunit and stearoyl-ACP desaturase), storage (concanavalin A-like lectin), defense/immunity (disease resistance RPP13-like, basic 7S globulin, MACPF domain-containing protein, thaumatin domain family, glucan endo-1,3-beta-glucosidase 12-like, O-glycosyl hydrolase family 17 and DUF231) and development in response to abiotic stimulus (chaperone protein dnaJ 20, glucomannan 4-beta-mannosyltransferase 9, ARM repeat superfamily and argininosuccinate lyase) were found to be relatively abundant in rhizome. Argininosuccinate lyase is essential for normal root elongation by arginine production besides ornithine accumulation through urea cycle under low CO_2_ availability [53]. Earlier, high expression of ARM family transcript has been reported specific in rhizome that promotes root growth [54]. Deep root elongation reduces the effects of environmental stresses and limits soil erosion. While, both rhizome and root perform mechanical and storage functions, the efficient growth and development of belowground system is not only associated with vegetative regeneration but also increases species survival under stressful environment. Therefore, each organ performs a specialized task in the life cycle of a plant, which is directly reflected in its proteome and deeply influenced by the environment.

### Metabolic pathway mapping and enrichment highlights functional differences between early and late developmental stages of *P. kurroa*

Plant development is divided into vegetative (early) and reproductive (late) phases. Generally, the vegetative phase starts with pollination and ends with the commencement of flowering, whilst the reproductive phase begins with appearance of flowering organs and stops with pollination [55]. The enrichment of photosynthetic and Calvin cycle pathways in the vegetative phase will likely increase carbon availability that results increased plant growth. As plant grows from vegetative to generative stage, there is an increasing mass of reproductive organ that does not perform photosynthesis [56]. In that phase, plants carry temperature dependent respiration rate and thus, enhancement in glycolytic process and pyruvate metabolism were noticed in late stage to produce energy reservoirs. Photosynthesis is intricately associated with carbon fixation, however carbon assimilation process found to be relatively enriched in generative phase. Higher carbon fixation rate permit photosynthesis even in low CO_2_ to maintain resource use efficiency [57]. Although, RuBisCo catalyzes the carboxylation of ribulose-1,5-bisphosphate (RuBP) in photosynthesis, but also competes for oxygenation in photorespiration, thereby decreases photosynthetic efficiency. Photorespiration was found to be significantly enriched in the reproductive stage of *P. kurroa*, however less information is available about the orchestration of photorespiratory cycle during developmental phases. An earlier study reported that photorespiratory pathway is activated under fluctuating light conditions to benefit photosynthetic carbon fixation through regulation of electron flow [58]. In vegetative organs, lipid breakdown and phosphatidylinositol signaling might overcome the detrimental effects of free fatty acids and reactive oxygen species (ROS) that generate adaptive potential to adverse conditions [59]. Furthermore, enhanced cysteine biosynthesis might associate with glutathione pool in order to balance redox status of the cell. In addition, this could elevate the level of sulphur containing amino acids and improve the nutritional value of vegetative organs in *P. kurroa*. Reproductive development is more sensitive to abiotic stress as compared to vegetative phase, predominantly during fruit and seed setting [60]. Enrichment of metabolic pathways was found to be 1.6 folds on log scale in reproductive stage compared to vegetative. These metabolic pathways synthesized specialized compounds mostly during stationary growth phase and not implicated in reproduction or plant development. The pivotal role of sucrose and starch metabolism in plant development and stress response is well established as the interruption of sucrose signaling and metabolism caused significant reproductive failure in abiotic stress conditions [61]. On the whole, this reflects early stage is primarily associated with growth and development by utilizing less energy through alternative pathways, whereas the late stage harvests more energy in secondary metabolic pathways to synthesize diverse compounds of plant defense. Additionally, with reference to particular adaptive response, both early and late stage plants respond to environmental fluctuations through distinct processes.

### Accumulation of stress-responsive proteins may provide *P. kurroa* adaptation to stress combinations at high-altitude

**Fig. 7:**
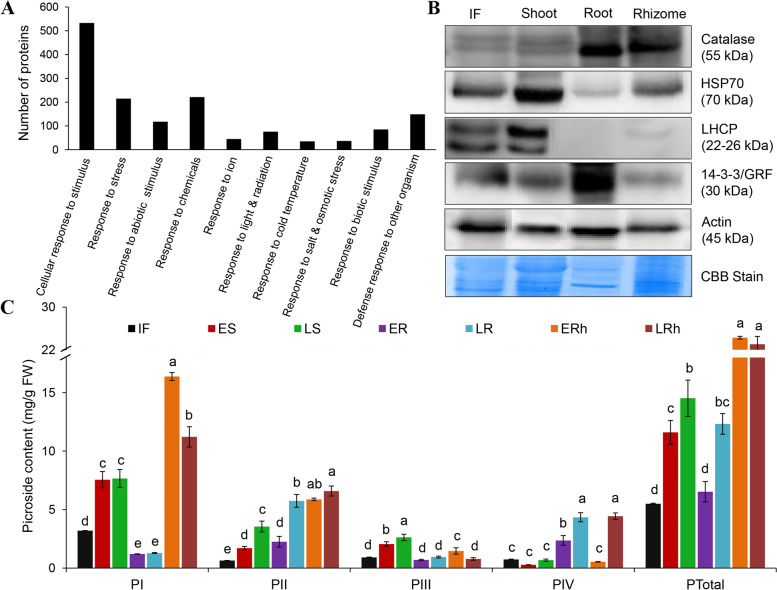
Over-representation of *P. kurroa* stress-responsive proteome compared with a customized reference of nine green plant species viz*. Z. mays, G. max, A. thaliana, S. tuberosum, T. aestivum, N. tabacum, P. sativum, O. sativa* and *H. vulgare*. **A** Number of *P. kurroa* stress-related proteins into diverse functional categories. Protein functions were predicted and categorized using AgriGO v. 2.0. **B** Validation of protein expression data of selected stress-responsive marker proteins in different *P. kurroa* organs using western blotting. Approximately, 25 µg of proteins from inflorescence, shoot, root and rhizome were separated by 12.5% SDS-PAGE, transferred to a PVDF membrane, and probed with antibodies against catalase, heat shock protein 70, type 1 chlorophyll a/b binding protein and 14-3-3 GRF. Actin was used as a loading control. The experiment was performed thrice with similar results. **C** Determination of picrosides content (PI-IV) in different organs at two developmental stages of *P. kurroa*. The abbreviations used are follows: IF, Inflorescence; ES, Early shoot; LS, Late shoot; ER, Early root; LR, Late root; ERh, Early rhizome; LRh, Late rhizome. Data are represented as the means ± S.D (*n* = 3) and different letters indicate statistically significant differences using one-way ANOVA followed by Tukey HSD post-hoc test (*p* < 0.05) for each variable autonomously in R v. 4.0.0

Our proteome map unravels many candidates responsive to abiotic and biotic stimuli, which are required for the synthesis of various regulatory/functionally defensive proteins and protective metabolites. These proteins accounted for 15.1% of the total identified proteome, which are of interest for *P. kurroa*, a potentially emerging model species for studying the adaptability to stress combinations prevailing at higher altitudes. Climate at high-altitudes are said to be harsh; specifically, low water and gases availability, fluctuating temperatures, high radiations, wind velocity and snow cover limit plant survival. Among functionally annotated stress-related proteins, response to stimulus (68.15%), chemicals (28.26%), stress (27.49%), other organism (19.05%), abiotic (15.08%), biotic (10.86%), light or radiation (9.71%), ions (5.75%), salt or osmotic stress (4.73%) and cold temperature (4.47%) were found to be over-represented in *P. kurroa* (Fig. 7A).

In response to alpine environment, *P. kurroa* could have some special stress-responsive proteins. Various classes of heat shock proteins (HSPs) including chloroplastic and mitochondrial localized, chaperonins and peptidyl-prolyl isomerase have been identified. These molecular chaperones facilitate protein folding, assist translocation and degradation of damaged ones under stress conditions. Additionally, heat shock factors were also observed that regulate the expression of these HSPs. To detoxify ROS and maintain redox homeostasis, the key enzymatic antioxidants like catalase, monodehydroascorbate reductase, dehydroascorbate reductase, glutathione peroxidase, glutathione reductase, glutathione S-transferase, 2-Cys peroxiredoxins and osmotin-like are evident in *P. kurroa* (Table S1). In brief, environmental stress is recognized by stress sensors that mediate signal transduction cascade to regulate gene expression or metabolic pathways imparting stress endurance to plants. Various stress signaling proteins (14-3-3, GF, calreticulin and calmodulin) and downstream components like kinases (casein, His, Ser/Thr, wall-associated, glycerol, receptor like, LysM domain, diacylglycerol, CLAVATA, farnesol, MAPK, CDPK and CIPK), phosphatases (Ser/Thr, inositol phosphate, phosphatidylinositol and lipid phosphatase), transcription factors (TFs) and channels/transporters were detected that play a crucial role in orchestration of stress responses in *P. kurroa*. The identified TFs such as zinc finger proteins, ERF107, ERF053, ERF118, AP2/ERF and B3 domain, WRKY, AIL5, ABI5, ONAC010, bHLH131, MYC2, NAC, bZIP, ERF1B, ERF027, RAP2-11, MYB3R3, MYB1R1 and AP2/EREBP among others were considered to be involved in the regulation of stress-related gene expression [62]. The ABA biosynthetic proteins like zeaxanthin epoxidase, aldehyde oxidase, 9-cis-epoxycarotenoid dioxygenase, molybdenum cofactor sulfurase and its downstream signaling signatures PYL, PP2C, ABFs along with chloroplastic receptor CHLH were caught attention. The identification of ABA-induced transporters/channels like ABCG, peptide/nitrate, two pore K^+^ channel, guard cell outward rectifying K^+^, Na^+^/H^+^ and cation-H^+^ exchanger shed light on ABA-mediated stress tolerance by regulating stomatal movements [63]. In addition to water regulation by aerial parts through stomata, belowground uptake is found to be enhanced by root growth through the involvement of vacuolar H^+^-pyrophosphatase that increases water retention and emphasizes organ-specific adaptation mechanisms. In continuation, diverse aquaporins were also detected that are key players of plant-water relations. Apart from this, a large number of ABC transporters have been identified including vacuolar, mitochondrial and multidrug resistance-associated proteins, which indicate the characteristics of heavy metals (HMs) tolerance that could be further explored for phytoremediation. In accordance, other observed transporters such as copper transport family, pleiotropic drug resistance protein, metal-nicotianamine YSL, heavy metal ATPase, Nramp family, MATE family, HM-associated isoprenylated protein and metal tolerance proteins are also considered to facilitate HMs tolerance [64].

**Fig. 8:**
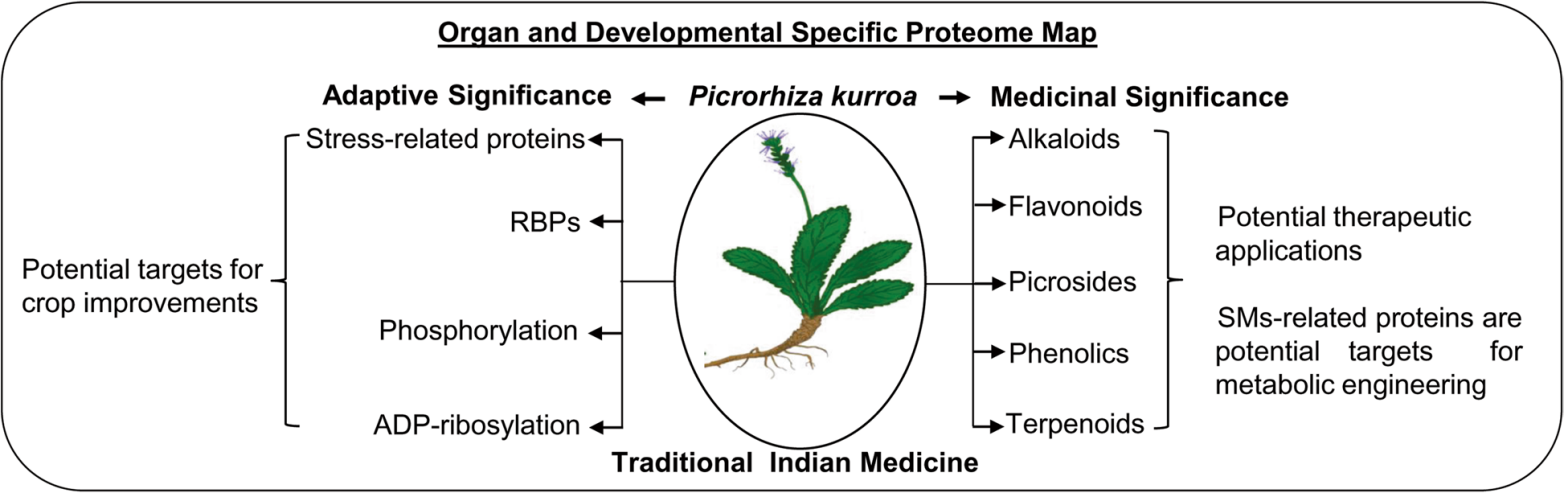
Organ and development dissected proteome map of *P. kurroa* reveals medicinal and adaptive significance. A quantitative analysis of proteins associated with stress-response and synthesis of secondary metabolites are potential targets for crop improvement and metabolic engineering, respectively

Proteins involved in metabolic processes were found to be the largest group and of significant interest for understanding the adopted basal metabolism of alpine herbs. Stress metabolism, which results changes in bioenergetics is well documented in *P. kurroa* proteome. Besides the identification of an array of photosynthetic proteins, photorespiratory enzymes were more evident (Table S1), which is considered as a defense line at molecular level against photooxidative damage. In addition, secondary metabolic enzymes were well observed in *P. kurroa* that lead to the formation of diverse secondary metabolites (SMs) having protective functions from stresses such as anthocyanins, flavonoids/phenolics, glucosinolates, volatiles, lignins, oxylipins and others. Moreover, several protective proteins were also enriched such as pathogenesis-related, LEA family, stress-related, PLAT-plant stress domain, thioredoxin, lipoxygenase and lactoylglutathione lyase that enable *P. kurroa* to resist several abiotic and biotic stress combinations at high-altitudes. Further, western blotting was performed to validate the expression of stress-responsive proteins in different organs (Fig. 7B; Fig. S1). Subsequently, four stress-related proteins, namely heat shock protein 70 (HSP70), catalase, LHCP and 14-3-3 were selected for expression analysis. The western blotting data was in agreement with our proteomic results and discrepancies were observed among organs. HSP70 was universally expressed in all four organs with more abundance in shoot. Catalase was highly expressed in root and rhizome, whereas 14-3-3 abundance detected more in root. Shoot showed highest abundance for LHCP followed by inflorescence, which was not detected in root. Therefore, we conclude that stress-responsive proteins provide adaptive significance to *P. kurroa* towards harsh environment and might be potential targets for crop improvement under adverse conditions (Fig. 8).

### Proteins associated with secondary metabolism may provide medicinal significance to *P. kurroa*

*P. kurroa* is enriched with many SMs in an organ-specific manner having diverse biological effects [7]. These SMs have thought to be key drivers of pharmacological actions, which provide the foundation for its use in the traditional and modern medicine system. Being a medicinal herb, *P. kurroa* proteome was found to be functionally enriched for small molecule metabolic process (289 proteins, *p* = 1.3E-23), generation of precursor metabolites (81 proteins, *p* = 3.5E-12), secondary metabolic process (35 proteins, *p* = 1.4E-08), secondary metabolite biosynthetic process (24 proteins, *p* = 7.9E-07), small molecule biosynthetic process (83 proteins, *p* = 3.7E-06) and terpenoid metabolic process (17 proteins, *p* = 5.6E-06). This revealed that proteins associated with SMs, especially phenolics, flavonoids, alkaloids, terpenoids, iridoids, glucosinolates and plant hormones were mostly identified in rhizome compared to other organs. At organ level, proteins linked with phenolics were predominant in rhizome, while those associated with terpenoids were greatest in the shoot. Terpenoid biosynthesis is an important function in *P. kurroa*, which is found to be operated mostly in the shoot by the involvement of both mevalonate and non-mevalonate pathways. Apart from this, several identified proteins were mapped on the unique KEGG metabolism revealing organ-specificity at pathway level such as proteins involved in α-linoleic acid metabolism, lipopolysaccharide biosynthesis, β-alanine metabolism, pantothenate biosynthesis, taurine and hypotaurine metabolism and isoquinoline alkaloid biosynthesis were exclusively identified in the rhizome. While, proteins play a key role in zeatin biosynthesis and methane metabolism were observed in root and inflorescence, respectively. However, proteins related to photosynthesis, sesquiterpenoid/triterpenoid biosynthesis and biotin metabolism were detected uniquely in the shoot.

Of particular interest, *P. kurroa* is known for its specialized metabolites i.e. picrosides. The picroside biosynthesis is associated with mevalonate, non-mevalonate, iridoid and shikimate/phenylpropanoid pathways. Two proteins of plastidic non-mevalonate pathway like 1-deoxy-D-xylulose-5-phosphate synthase and 4-diphosphocytidyl-2-C-methyl-D-erythritol kinase were identified only in the shoot, which might synthesize picrosides. The cytosolic mevalonate pathway proteins such as acetyl-CoA acetyltransferase, 3-hydroxy-3-methylglutaryl-coenzyme A reductase 2 and acetoacetyl-CoA thiolase 1 were observed in rhizome and shoot, respectively. In addition, proteins belong to shikimate/pheylpropanoid pathways such as shikimate O-hydroxycinnamoyltransferase, phospho-2-dehydro-3-deoxyheptonate aldolase 2, chorismate mutase, arogenate dehydratase were detected in shoot and rhizome. Furthermore, phenylalanine ammonia-lyase, a key enzyme involved in secondary metabolism and phenolics accumulation was found in the rhizome, which might produce a variety of SMs. While, the main enzyme of iridoid pathway, heterodimeric geranylgeranyl pyrophosphate synthase was identified in all organs, suggesting all *P. kurroa* organs contributed in picroside biosynthesis. Thus, to gain more insights into metabolic differences among *P. kurroa* organs at different developmental phases, the marker compounds viz. picrosides (I-IV) were quantified using UHPLC-PDA system (Fig. 7C; Fig. S2). Among four picrosides, PI content was found to be highest followed by PII. Additionally, a significant variation was found to be associated with both organ and developmental stages. The highest content of PI was observed in rhizome followed by shoot, inflorescence and root. The maximum level of PII was observed in rhizome, which slightly reduced in root followed by shoot and inflorescence. However, the quantity of PIII and PIV was less, while the greatest amount was detected in shoot and root, respectively. The picroside content also varies significantly with the stage of development. In rhizome, PI and PIII decreased, while PII and PIV increased significantly at the late stage compared to an early phase. Also, a significant enhancement was noticed for PII and PIV in root and PII and PIII in shoot at the late stage of development. As a result, total picroside content elevated significantly at the generative phase in root and shoot, while no significant change was observed in rhizome. Overall, total picroside content was highest in rhizome followed by shoot compared to other organs, which is in line with our proteomic data. These secondary metabolisms related proteins would be potential targets for enhanced specialized metabolites production through metabolic engineering (Fig. 8).

## Conclusions

This study is navigated towards the systematic investigation of whole proteome in high-altitude medicinal herb, *P. kurroa*. The use of 1D-GE and nanoLC-MS/MS enabled us to achieve the deep proteome coverage with 5186 protein accessions enriched for 359 GO terms, when compared with green plants so far. The quantitative analyses of sub-proteomes across four major organs with regard to biological functions and protein abundance provide a comprehensive map of organ-specific and shared functions. Also, pathway-centric functional mapping at key developmental stages highlights the distinct adaptive processes to alpine environmental perturbations. Deep analysis of stress-responsive biological functions provides several candidate proteins involved in multiple stress tolerance that could be potential targets for crop improvement*.* Additionally, proteomic analysis led to the detection of several proteins linked with secondary metabolism for the synthesis of picrosides, flavonoids, alkaloids, phenolics and terpenoids that could offer novel opportunities of metabolic engineering for enhanced SMs production. *P. kurroa* proteome profiling will enrich the proteome database and presents a good groundwork for further studies with an ultimate goal of complete understanding of complex molecular networks for specialized metabolites production and combinatorial stress tolerance.

## Methods

### Plant sampling

*P. kurroa* was collected from Rohtang pass in Pir Panjal range of Western Himalaya, India (32°23′30''N, 77°15′24"E; 3400 masl) in July 2017 and 2019 during an extended noon period. India’s Biological Diversity Act 2002 permits bonafide Indians to access biological resources for scientific research [65]. The voucher specimen has been deposited in an internationally recognized herbarium of CSIR-IHBT, Palampur with acronym ‘PLP’. The identification of plant specimen was performed by taxonomist of our institute and complete information of voucher specimen like scientific name, *Picrorhiza kurroa* Royle ex Benth.; common name, Kadu; family, Plantaginaceae; place of plant collection, Rohtang Pass was recorded under specimen no. 15384 as described previously [7]. Four different organs viz*.* shoot, root, rhizome and inflorescence at two developmental stages i.e. vegetative (before flowering) and reproductive (after flowering) were collected, instantly frozen in liquid nitrogen and stored at -80 ºC until use. To construct *P. kurroa* proteome map, at least eight independent plants of each growth stage (vegetative and reproductive) were used for dissecting morphologically differentiated four plant organs.

### Protein extraction and determination

Protein extraction from different organs (shoot, root, rhizome and inflorescence) at key developmental stages was performed as explained by Kumar et al. [66]. Approximately, 2 g of each frozen sample was ground to fine powder in liquid nitrogen and transferred into prechilled centrifuge tube having 10 ml of homogenization buffer [Sucrose (40%), 1 mM EDTA (pH 7.5), 50 mM HEPES–KOH (pH 7.5), Triton X-100 (1%), 1 mM PMSF, β-mercaptoethanol (1%) and 10 mM ascorbic acid]. The homogenate was vortexed and stirred with tris-equilibrated phenol (pH 8.0) on rocker for 30 min prior to centrifugation at 5000 g for 15 min. The phenol phase was separated and precipitated overnight with 0.1 M ammonium acetate in methanol. Through centrifugation at 10,000 g for 15 min, protein pellets were recovered, cleansed with 80% acetone twice, air dried and solvated in rehydration buffer [8 M urea, CHAPS (2%) and 2 M thiourea]. All steps were conducted on ice or at 4 °C. Protein concentration was determined by Bradford method with BSA as a calibration standard [67].

### SDS-PAGE and nanoLC-MS/MS

Approximately, 25 µg of each protein sample were dissolved in sample buffer [0.25 M Tris–Cl (pH 6.8), glycerol (50%), SDS (10%), 0.5 M dithiothreitol (DTT), bromophenol blue (0.25%)] and resolved by SDS-PAGE on 12.5% polyacrylamide gels (Biorad, India). Subsequently, fractionated proteins were visualized with CBB and each sample lane was excised into eight fractions. In-gel digestion was performed according to a standard protocol [68]. The excised gel pieces were destained with 100 mM NH_4_HCO_3_ and acetonitrile (ACN) followed by washing with water thrice. The gel pieces were reduced with 10 mM DTT in 100 mM NH_4_HCO_3_ at 60 ºC for 45 min, alkylated with 50 mM iodoacetamide (IAA) in 100 mM NH_4_HCO_3_ at RT in dark for 20 min followed by washing with 100 mM NH_4_HCO_3_. Trypsin digestion was carried out with 10 ng/µL trypsin (Promega, India) in 50 mM NH_4_HCO_3_ at 37 °C for 12–16 h. Peptides were extracted with 0.1% trifluoroacetic acid  in H_2_O:ACN (50:50) and lyophilized (Labconco, USA).

For nanoLC-MS/MS, tryptic peptides were redissolved in 0.1% HCOOH/ACN and loaded on BEH C18 column (1.7 μm, 75 μm × 200 mm; Waters, India) equilibrated for 3 min with 0.1% HCOOH having a flow rate of 5 μL/min. NanoLC-MS/MS was performed with HPLC chip coupled to a 6560 Q-TOF (Agilent Technologies, USA). A linear gradient of 75 min was employed for peptide separation by a series of 1200 nano pump (Agilent Technologies, Santa Clara, USA) through two solvent system comprises A; 0.1% HCOOH in water and B; 0.1% HCOOH in acetonitrile. The gradient program starts with 5–8 min, 3–15% B; 8–50 min, 15–45% B; 50–55 min, 45–90% B, hold for 5 min and finally resume within 1 min. The subsequent parameters were placed in the acquisition mode: MS scan range (m/z) and rate (spectra/sec), 50–1700 and 3; MS/MS scan range (m/z) and rate (spectra/sec), 50–1700 and 1; collision energy, a slope of 3.6 V and an offset of 4.8 V. While, the following criteria were specified for precursor selection: threshold (Abs), 5000; max. precursor per cycle, 3; threshold (Rel. %), 0.010; active exclusion excluded after 2 spectra and released after 0.5 min; target (counts/spectrum), 25,000; preferred charge state, 2, 3, > 3. The instrument parameters were set at positive ionization mode, Gas Temp (°C), 325; Gas Flow (l/min) 6; capillary voltage, 2000 V and skimmer, 65 V.

### Protein identification and quantification

Raw data (.d files) were analyzed using Spectrum Mill Proteomics Workbench software (Agilent Technologies, USA) that extracts spectra, MS/MS searches for spectra and validates peptides or proteins in the database. The default extraction parameters used were: MH^+^ precursor range (Da), 600–4000; scan time range (min), 0–300; time and mass window for merging scans by spectral similarity and RT and *m/z*, ± 60 s and ± 1.4 m*/z*. MS/MS search criteria were modified as: database, NCBInr; species, plants; fixed modification, carbamidomethylation (C); match filtering, disabled; mass type, monoisotopic; min. matched peak intensity (%), 50; max. ambiguous precursor charge, 3; precursor mass tolerance (ppm), 20; product mass tolerance (ppm), 50; search mode, identity with enabled calculate reversed database scores. MS/MS auto validation was performed on peptide mode with FDR < 1%. The results were shown in “protein/peptide summary report” after filtered by peptide score threshold ≥ 3.0 and percentage scored peak intensity (% SPI) threshold ≥ 60%. An intensity-based absolute quantification algorithm (iBAQ) was used to calculate the protein abundance [69]. The protein intensity was first calculated by Spectrum Mill software as a total of the entire identified peptide intensities. Then, protein intensity was divided by the theoretically observable peptides number (evaluated by in silico protein digestion using MS digest tool and all tryptic peptides length between 6–30 amino acids were considered). The resulted intensity was shown as “absolute iBAQ intensity” (iBAQ). For sample to sample comparison and data normalization, the “relative iBAQ intensity/fraction of total iBAQ” (FOT-iBAQ) of each protein was assessed by dividing the absolute iBAQ intensity by a total of absolute iBAQ intensities of the entire proteins in the sample. Finally, FOT-iBAQ value was multiplied by 10^5^ to get “intensity-based fraction of total” (iFOT) value for simple visualization of low abundant proteins [70]. The estimated iFOT represented the abundance of protein in each organ.

### Bioinformatics and statistical analysis

A circular proteome map of *P. kurroa* was generated for four morphologically differentiated organs using Circos (v.0.69–9) [71]. The assignment of protein functions was based on (GO) categories of nine plant species (identified for *P. kurroa* total proteome) covering *Z. mays*, *G. max*, *A. thaliana*, *S. tuberosum*, *N. tabacum*, *T. aestivum*, *P. sativum*, *O. sativa* and *H. vulgare*. Enrichment analysis of GO categories was performed in AgriGO (v.2.0) using Fisher’s exact test (*p* < 0.05) [72]. Over and under-representation of GO categories was computed for total proteome (5186 identified proteins) as compared to a customized reference proteome, which includes aforementioned nine plant species. The proteome of each organ was also compared to total proteome for assessing organ-specific enriched GO terms (*p* < 0.05). PCA and correlation study were assessed in R (v.4.0.0) based upon the protein abundance. Hierarchical clustering analysis was also implemented in R (v.4.0.0) for those proteins present in at least two organs and an optimum number of clusters were obtained based upon Silhouette index (0.66). KEGG pathway mapping software iPATH (v.3.0) was used to visualize the differences between developmental stages at metabolic pathway level [73]. Also, pathway enrichment was performed for protein groups of both stages against aforementioned plant species using ShinyGO (v.0.61) [74]. ModPred tool was used to identify peptides containing PTMs with high confidence (false positive rate of 0.01) [75]. Next, domain information was fetched for all identified proteins from Pfam database [76]. Notable biophysical properties of RBPs such as protein length, isoelectric point, hydrophobicity, disordered regions were assessed using ProtParam (https://web.expasy.org/protparam), isoelectric point calculator, protein GRAVY and IUPred2A, respectively [77–79]. The comparative analysis of biophysical properties between RBPs and non-RBPs was performed using Kolmogorov–Smirnov test at 1% level of significance. Also, a motif enrichment analysis was performed for 1272 putative RBPs using DREME software (v.5.1.1) with a threshold E-value of 0.05 [80].

### Western blotting

To validate the proteins expression, the total extracted proteins from four different organs viz*.* inflorescence, shoot, root and rhizome were used for immunoblotting. Briefly, 25 µg of protein were resolved onto 12.5% SDS-PAGE and transferred onto 0.45 µm PVDF membrane (Amersham^TM^Hybond™, GE Healthcare, Germany) using a wet transblot system. Afterwards, membranes were blocked with 2.5% non-fat dry milk in TBST (10 mM Tris–HCl, pH 7.5; 15 mM NaCl; 0.05% tween 20) at 4 ºC overnight, washed thrice and probed with rabbit polyclonal antibodies raised against catalase (Agrisera AS09 501), HSP70 (Agrisera AS08 371), LHCA1 (PS1 chlorophyll a/b-binding protein, Agrisera AS01 005), 14-3-3 GRF (Agrisera AS12 2119) and actin (Agrisera AS13 2640) followed by incubation with horseradish peroxidase (HRP)-conjugated goat anti-rabbit secondary antibody (Agrisera, AS09 602) for 1 h at RT. Blots were developed using ECL™ Prime detection reagents (GE Healthcare, Germany) and visualized under an Azure c300 Chemiluminescent Imaging Biosystem (California, USA).

### Determination of picrosides content in *P. kurroa*

Approximately, 100 mg fresh weight sample was extracted in 70% methanol and quantitative analysis of picrosides was performed using UHPLC-PDA system (Agilent, Santa Clara, USA) as described previously [7].

## Supplementary Information

**Additional file 1: Table S1.** List of proteins identified through 1D-GE-nanoLC-MS/MS in different organ and developmental dissected samples of *P. kurroa*.

**Additional file 2: Table S2.** List of protein accessions with their calculated relative abundance (iFOT) in each of the organ proteomes.

**Additional file 3: Table S3.** Gene ontology enrichment analyses of whole proteome and organ proteomes using AgriGO (Fisher's exact test and Bonferroni adjustment, *p* < 0.05). GO categories type: C, Cellular component; F, Molecular function; P, Biological process.

**Additional file 4: Table S4.** Five protein clusters were generated by hierarchical clustering analysis (silhouette index > 0.66) comprised of protein accessions, their description along with calculated Z-scores in each of the organ.

**Additional file 5: Table S5.** Proteome-wide analysis of protein domains and screened RNA-binding domains (RBDs) using Pfam database. A list of *P. kurroa* putative RNA-binding proteins (RBPs) and their motif enrichment using DREME software (E-value < 0.05).

**Additional file 6: Table S6.** List of different post-translational modifications observed at peptide level using ModPred. Also, detailed list of ADP-ribosylated and phosphorylated peptides and their protein identifiers along with modified residues.

**Additional file 7: Fig. S1.** Western blots and gel images in raw form for all the organ samples. The abbreviations used are as follows: IF, Inflorescence; S, Shoot; R, Root; Rh, Rhizome; M, Marker lane (10–250 kDa).

**Additional file 8: Fig. S2.** UHPLC chromatograms of picrosides extracted in 70% methanol at 270 nm. The abbreviations used are: IF, Inflorescence; ES, Early shoot; LS, Late shoot; ER, Early root; LR, Late root; ERh, Early rhizome; LRh, Late rhizome; PI, Picroside I; PII, Picroside II; PIII, Picroside III; PIV, Picroside IV.

## Data Availability

All the data are presented in this manuscript and in the supporting files are available via ProteomeXchange with identifier PXD030263.

## Abbreviations

ACN: Acetonitrile
CBB: Coomassie brilliant blue
DTT: Dithiothreitol
FDR: False discovery rate
GO: Gene ontology
IAA: Iodoacetamide
iBAQ: Intensity-based absolute quantification
iFOT: Intensity-based fraction of total
LC: Liquid chromatography
MS: Mass spectrometry
1D-GE: One-dimensional gel electrophoresis
PI: Picroside I
PII: Picroside II
PIII: Picroside III
PIV: Picroside IV
PCA: Principal component analysis
PTMs: Post-translational modifications
Q-TOF: Quadrupole-time of flight
RBPs: RNA binding proteins
SDS-PAGE: Sodium dodecyl sulphate–polyacrylamide gel electrophoresis
SMs: Secondary metabolites
SPI: Scored peak intensity
2D-GE: Two-dimensional gel electrophoresis
