# Supplementary material for: In-depth assembly of organ and development dissected Picrorhiza kurroa proteome map using mass spectrometry
Source: BMC Plant Biol. 2021 Dec 22;21:604. doi: 10.1186/s12870-021-03394-8 (PMC8693493; doi:10.1186/s12870-021-03394-8)
Supplement: Supplementary file 6 — Additional file 6: Table S6. List of different post-translational modifications observed at peptide level using ModPred. Also, detailed list of ADP-ribosylated and phosphorylated peptides and their protein identifiers along with modified residues. [file 12870_2021_3394_MOESM6_ESM.docx]

**Table S6. Number of different post-translationally modified peptides using ModPred**

| **Modification type** | **Number of Peptides** |
| --- | --- |
| Proteolytic_cleavage* | 519 |
| Amidation* | 431 |
| Phosphorylation | 95** |
| ADP-ribosylation | 89** |
| Carboxylation | 58 |
| Ubiquitination | 54 |
| Methylation | 53 |
| Acetylation | 50 |
| SUMOylation | 47 |
| PUPylation* | 28 |
| O-linked_glycosylation | 24 |
| N-linked_glycosylation | 17 |
| Hydroxylation | 12 |
| Pyrrolidone_carboxylic_acid | 6 |
| GPI_anchor_amidation | 5 |
| Palmitoylation | 4 |
| N-terminal_acetylation | 1 |
| Sulfation | 1 |

* Excluded PTMs from analysis using ModPred. Proteolytic cleavage is not a PTM according to conventional definition. PUPylation is exclusively a prokaryotic PTM and amidation can act on any amino acid, so prediction showed high score biasing any statistical test (Pejaver et al., 2014).

** ADP-ribosylated and phosphorylated peptides and their protein identifiers along with modified residues.

| NP_001169421.1 | R14 | ADP-ribosylation | ASVGLARVSGVARPLMRLVTMSRMP |
| --- | --- | --- | --- |
| CAA68141.1 | R437 | ADP-ribosylation | RQVTVDRPDVAGRVKILQVHSRGKA |
| XP_006342112.1 | R429 | ADP-ribosylation | RQVTVDRPDVAGRVRILQVHSRGKA |
| AFW64540.1 | R237 | ADP-ribosylation | VSKFKVASLLSPRLEITEDSIVLRR |
| XP_008650312.1 | R203 | ADP-ribosylation | SSAASGELGPGPRATPALGRPLGRP |
| XP_008650312.1 | R210 | ADP-ribosylation | LGPGPRATPALGRPLGRPPRAAPGS |
| NP_563683.1 | R276 | ADP-ribosylation | SEKVVEATKDVSRLKLEGNDGMGGG |
| NP_173208.1 | R107 | ADP-ribosylation | SVIMTDRITGNPRGFGFVTFADSAV |
| AFW56190.1 | R760 | ADP-ribosylation | RSDGGMRLGFVERLDIVLDVSMAME |
| XP_008661149.1 | R175 | ADP-ribosylation | MEPQLSPFSISGRSRLIALLSG*** |
| ACF84171.1 | R81 | ADP-ribosylation | RCPRCAARVPPLRGKAAGAATVGSG |
| XP_003532367.1 | R691 | ADP-ribosylation | KVDDDVASPDVDREIKTPSRPTVSQ |
| NP_001149897.1 | R243 | ADP-ribosylation | TDDDDVLDDPVSRKSKGSSSYSKQN |
| XP_006358894.1 | R527 | ADP-ribosylation | NIEAAADLDLGERALPAVVDKSASL |
| AAO00820.1 | R24 | ADP-ribosylation | GYFGASSSVVPLRALFDHGFDLNMP |
| XP_006358815.1 | R232 | ADP-ribosylation | PELIESLAEGDFRKNIPRFKPENFE |
| AFW77687.1 | R348 | ADP-ribosylation | VRAMGNGALEVGRLRSGLRAAVASC |
| XP_006594337.1 | R115 | ADP-ribosylation | YVTETVESLELKRVLGNDSIPKRLP |
| NP_001145826.1 | R231 | ADP-ribosylation | LGEVGGLRVFPLRGLMKGGKERDVK |
| XP_008667875.1 | R475 | ADP-ribosylation | GAAGRWGEVASTRKEIRSRSTRKTP |
| XP_006573900.1 | R21 | ADP-ribosylation | LGNLVESIKSKVRSLKPKKGKKPYM |
| XP_006354560.1 | R18 | ADP-ribosylation | LLPSTNVTLSKSRPKYPLSPSPFHP |
| ACG47669.1 | R56 | ADP-ribosylation | LISTAKSVASPGRGILAIDESNATC |
| XP_003556085.1 | R296 | ADP-ribosylation | DHGISPSQSPEGRGKKSPQNGHGSS |
| NP_850630.1 | E43 | ADP-ribosylation | GGGSGGPRFNVKEGDAKGDASVRFA |
| NP_182083.1 | R407 | ADP-ribosylation | VMEWIGSEIKKERPSNNKEWINNGD |
| XP_008664958.1 | R259 | ADP-ribosylation | LEAKLVAAMVDKRELQAEVAAKKKE |
| NP_001170197.1 | R115 | ADP-ribosylation | APAAASPYCALLRAALFGLDTPDRV |
| NP_001143319.1 | R618 | ADP-ribosylation | YGSVVDVATLGPRPSKGVEVEPIRI |
| XP_006592862.1 | R499 | ADP-ribosylation | EKVIVHDTAISGRPNVAEMSVNAAI |
| XP_006583646.1 | R61 | ADP-ribosylation | SMISKIVSSVPPRKNEVEEFLNSEI |
| NP_198085.1 | R300 | ADP-ribosylation | DMNEIVESLTKEREGLRGQVVGLEK |
| NP_001145394.1 | R71 | ADP-ribosylation | GHDASSNTQPASRVNGQKPPPALEA |
| XP_006338159.1 | R636 | ADP-ribosylation | LVDTFSEIVSSKRPRNGFCCKLWH* |
| AFW63270.1 | R85 | ADP-ribosylation | TSHVRPSLPGPGRDRFGAVRRAPGG |
| NP_173910.1 | R439 | ADP-ribosylation | NFGVKVADFGLSRLLPSDVTHVSTA |
| AFW65415.1 | R129 | ADP-ribosylation | EVSGTRSGGGSGRGVGAEEAAPAPP |
| XP_008653654.1 | R37 | ADP-ribosylation | EVGAGAGVAEPGRGGAGTGRSSRGS |
| XP_003537372.1 | R29 | ADP-ribosylation | ERKISINNGGGGRALLLTPPTSSSS |
| AAL77115.1 | R13 | ADP-ribosylation | MEPLSIFQLSVPRGEFYEPPPSSEH |
| NP_178463.1 | R395 | ADP-ribosylation | IFFDEADVVACKRGDESSSNSSTVG |
| XP_003517765.1 | R137 | ADP-ribosylation | ADEELSEYRKDSRGAAPESSFAARL |
| NP_001167753.1 | R3 | ADP-ribosylation | **********MERETIDASLQNSIS |
| XP_006357155.1 | R383 | ADP-ribosylation | GLTFGIVPFVSKRSLGVISGMTGSG |
| XP_008673128.1 | R34 | ADP-ribosylation | RRIPSTAISARLRPEGTFSWLPVSC |
| XP_006360749.1 | R182 | ADP-ribosylation | EASNECSKLAVERTLNYECNDSPST |
| XP_006587014.1 | R42 | ADP-ribosylation | ISLSKQMEVKGIRANFVTLNILINC |
| NP_567356.1 | R332 | ADP-ribosylation | FINTVETLMAKSRVKAKMKRSKSRA |
| BAB01086.1 | R147 | ADP-ribosylation | LQEQVMLALVTIRKLSLQGQCLTIR |
| XP_008647562.1 | R26 | ADP-ribosylation | RASSLSSSAPALRGASLLRGDGPIG |
| XP_006582789.1 | R375 | ADP-ribosylation | GERQSARDLVQPRSQMEKVPVGDVP |
| DAA36559.1 | R171 | ADP-ribosylation | ISLNTFGSPAPIRSVQPRQDVVKHR |
| NP_001142121.1 | R36 | ADP-ribosylation | APALGKSKAVPGRADAMNRRAPLGD |
| DAA39943.1 | R19 | ADP-ribosylation | SKSSVAASPADLRSAMLLSDANARS |
| NP_001119051.1 | R121 | ADP-ribosylation | DESEDDETRANFRPNPVIIPKKNAR |
| XP_006591063.1 | R451 | ADP-ribosylation | HSGVKVIIEGNLRLGNQPVSSPSPM |
| ACL53146.1 | R503 | ADP-ribosylation | LEDFVNSVGDSFRPVGISVAEKTDE |
| AFW73850.1 | R475 | ADP-ribosylation | KDSRVVSSSIPSRGVSPRRRLASDG |
| AFW73850.1 | R481 | ADP-ribosylation | SSSIPSRGVSPRRRLASDGVDTMCR |
| NP_201348.1 | R154 | ADP-ribosylation | STSRPSIHKVQPRSEAVDFTTSLNI |
| NP_001168245.1 | R129 | ADP-ribosylation | LDLRHPDHVVVGRPPYLAPDHASPS |
| NP_178968.1 | R162 | ADP-ribosylation | HKKNISSKPVGLRRSKSDTSSMVDS |
| DAA45548.1 | R118 | ADP-ribosylation | DGAVIRCGGKPARRRPSGEKERSET |
| XP_003540504.1 | R355 | ADP-ribosylation | IFLSADLFNAGIRPAINVGISVSRV |
| XP_003521462.1 | R64 | ADP-ribosylation | CGSFQVKCSAISRKDPFLDLHPEVS |
| XP_003535387.1 | R447 | ADP-ribosylation | VSSISSKSSRKSRGDLSQKSSRKSS |
| NP_001148233.1 | R132 | ADP-ribosylation | LEDGTVFDSSYKRGKPLTFRVGVGE |
| NP_196416.1 | R502 | ADP-ribosylation | QRAVPLVVHPKPRLAPNVYGLGSG* |
| AAM62864.1 | R265 | ADP-ribosylation | RELSIAEVEVPGRKPLVFATSHLES |
| XP_003541044.1 | R194 | ADP-ribosylation | VDPEKAMKEPLSRKVPIPSGRLSPY |
| NP_177885.1 | R74 | ADP-ribosylation | GATKKVAKGKSPRKTTPKKCATKNG |
| XP_006358852.1 | R296 | ADP-ribosylation | KESLIENVFKPNRKTPVA******* |
| XP_003540680.1 | R858 | ADP-ribosylation | KTKQDSSFEKVKRAAVSTLAAAAVK |
| XP_008647168.1 | R82 | ADP-ribosylation | PASSPARRAPLPRLGGPSLRDAPVR |
| XP_008647168.1 | R89 | ADP-ribosylation | RAPLPRLGGPSLRDAPVRPPCLGCG |
| NP_001159313.1 | R65 | ADP-ribosylation | DSDGEGAVGSGKRVRGVGGDGSATK |
| XP_006600846.1 | R353 | ADP-ribosylation | FNMMLSPSKEESRPVKSQPSRASSR |
| XP_008649658.1 | R364 | ADP-ribosylation | VETTDYLVLPPDRELSTDGYMELND |
| DAA53072.1 | R123 | ADP-ribosylation | SVGTDARPSTGGRLNGCSASLRKRH |
| XP_008656556.1 | R166 | ADP-ribosylation | AIGQHSNQTNKIRSYPHVDLKLVEG |
| XP_006348624.1 | R52 | ADP-ribosylation | NMNESDDVNTDSREKPSTSKATSVS |
| XP_006339502.1 | R169 | ADP-ribosylation | KEKILAAENAKLREKFGGLQQRETP |
| DAA58192.1 | R143 | ADP-ribosylation | LRARARAMEPVSRKRKGAPPVSATR |
| XP_006594217.1 | R165 | ADP-ribosylation | DVSENSEIDVGFRPSPTVASPGLKR |
| XP_006349771.1 | R927 | ADP-ribosylation | SRPPSGPAAPSSRPPSGPAAPMNCS |
| XP_006352274.1 | R136 | ADP-ribosylation | TPSTGSITPGPKRAKGRPPGSGWKQ |
| AAL34929.1 | R1449 | ADP-ribosylation | PRPRAPTVGAGPRPTAPISHGPRLP |
| XP_003535960.2 | R628 | ADP-ribosylation | VSKSPRKKSVAKRSLGSKPKVGATA |
| XP_006591261.1 | R103 | ADP-ribosylation | GHVIMLDVPGLKRDEIKIEVEGNRV |
| AFW79629.1 | T26 | Phosphorylation | TTPSSRARSTTSTPSPPKGHHRLQI |
| XP_003542773.1 | S67 | Phosphorylation | FERYLRPTSPPLSPSATTRRRSPSD |
| CDM83721.1 | S920 | Phosphorylation | SGDTGKLSERSGSPISSPAKTSVRE |
| CDM83721.1 | S924 | Phosphorylation | GKLSERSGSPISSPAKTSVRENSIQ |
| CDM83721.1 | S934 | Phosphorylation | ISSPAKTSVRENSIQESGVRRESRS |
| XP_003556250.1 | S16 | Phosphorylation | SRQKFSNQDSSLSPTAARSREWDGP |
| ACR34453.1 | S42 | Phosphorylation | ENLGNLLVPQRGSPR********** |
| XP_006578900.1 | S24 | Phosphorylation | VKKVFKSSSSKDSPLPEKKKENNKE |
| XP_008654081.1 | T20 | Phosphorylation | RRRAAATAAAVATPSELPPAGPRAS |
| NP_001235794.1 | Y463 | Phosphorylation | REFKANKISEEEYVKSIKEEIRKVV |
| AFW79629.1 | S28 | Phosphorylation | PSSRARSTTSTPSPPKGHHRLQIWR |
| XP_003542773.1 | S63 | Phosphorylation | VMAIFERYLRPTSPPLSPSATTRRR |
| NP_001144444.1 | S26 | Phosphorylation | TAAAVPDLSLHISPPSPPPAGGVEA |
| XP_008650312.1 | T205 | Phosphorylation | AASGELGPGPRATPALGRPLGRPPR |
| XP_006346045.1 | S180 | Phosphorylation | DPPQLSDLVPDASPEGKPKDEVEDD |
| AFW60786.1 | S307 | Phosphorylation | ISDDVSMMPLGVSPSPSSKLRERVP |
| AFW60786.1 | S309 | Phosphorylation | DDVSMMPLGVSPSPSSKLRERVPEE |
| XP_006342827.1 | S242 | Phosphorylation | FEADLEMGVRVSSPAIQSRSPATRL |
| XP_006342827.1 | S249 | Phosphorylation | GVRVSSPAIQSRSPATRLMSLRRIL |
| XP_006595069.1 | T332 | Phosphorylation | VATKFDSKPEASTPRRRSISTEKFL |
| XP_006595069.1 | S337 | Phosphorylation | DSKPEASTPRRRSISTEKFLSKEES |
| XP_006595069.1 | S339 | Phosphorylation | KPEASTPRRRSISTEKFLSKEESRI |
| DAA36324.1 | S1387 | Phosphorylation | QAKKHGRKGATLSPVTTEALRRSSR |
| XP_003530813.1 | T1122 | Phosphorylation | SGSNLKEGSNASTPEFRRSRSTPRG |
| XP_008661149.1 | S168 | Phosphorylation | AVKSMRKMEPQLSPFSISGRSRLIA |
| NP_174598.1 | S185 | Phosphorylation | NRLEARSFIRRESPYQGISMFELNK |
| XP_003555627.1 | T163 | Phosphorylation | GSPASVPPETSLTPPSSIPIKVSSN |
| AAB41023.2 | S421 | Phosphorylation | VSSGSEDDERDDSFEFDDKEKLREK |
| XP_008660976.1 | S22 | Phosphorylation | TFPVRRGERKLVSPSRPTPYEFKML |
| XP_008660976.1 | T27 | Phosphorylation | RGERKLVSPSRPTPYEFKMLSDIDD |
| DAA59491.1 | S289 | Phosphorylation | KQGVKLVDPPAPSPVPSSEPPPSPS |
| XP_006361259.1 | S86 | Phosphorylation | SLIEEEDEVEMESPIARVREKRIRR |
| XP_008673461.1 | T19 | Phosphorylation | LPRPAAAVASTSTPDIVAELGRVLS |
| NP_180393.1 | S659 | Phosphorylation | LAVNGGKYTRSGSDSGMMSSIQKPV |
| NP_001237556.1 | S112 | Phosphorylation | IVRFSTVIHERGSPETLRDPRGFAV |
| XP_003556085.1 | S288 | Phosphorylation | YHKERGRTDHGISPSQSPEGRGKKS |
| XP_003556085.1 | S292 | Phosphorylation | RGRTDHGISPSQSPEGRGKKSPQNG |
| XP_008665099.1 | T38 | Phosphorylation | SLLERTFLLGPGTPPRALLDGGRVR |
| XP_008668683.1 | S134 | Phosphorylation | HINIVPFKFDTPSPDDMVTTGLKSS |
| ACG48584.1 | T276 | Phosphorylation | GVATPVSITTSTTPSGAARSTPLRP |
| XP_008665222.1 | T217 | Phosphorylation | IASAVGVNEKKRTPSSPTHSMKMPS |
| XP_006363031.1 | T273 | Phosphorylation | EGQDATEIKKLETPTLSASAKSPPS |
| XP_006363031.1 | S282 | Phosphorylation | KLETPTLSASAKSPPSNSIIKQRSS |
| XP_006593875.1 | S48 | Phosphorylation | VHVFRRDDDSTSSPSEPPSSSDPSV |
| ACN35930.1 | S28 | Phosphorylation | NEMTRRTLNRRGSVHRRRGRGGGIA |
| XP_008652753.1 | S24 | Phosphorylation | IVISSPQGAVISSPPSSSSLRLVPL |
| XP_008660850.1 | S19 | Phosphorylation | PRPTPRRAPPPASPAAPPLSSPSPA |
| XP_008660850.1 | S26 | Phosphorylation | APPPASPAAPPLSSPSPAPRPVSPG |
| XP_008660850.1 | S27 | Phosphorylation | PPPASPAAPPLSSPSPAPRPVSPGS |
| XP_008660850.1 | S29 | Phosphorylation | PASPAAPPLSSPSPAPRPVSPGSRA |
| XP_003524494.1 | Y118 | Phosphorylation | EQGKNGSNDADNYSMFSTRTLASEK |
| XP_006354471.1 | T869 | Phosphorylation | GSLSPLQRTKIRTPLGSNGVQKGSK |
| NP_568633.2 | S1082 | Phosphorylation | IETQPRTIEKDLSPISITQPKNRSP |
| NP_001170159.1 | S176 | Phosphorylation | DAPPGLLRCRRRSPSGEATAPWIRG |
| NP_001131830.2 | T228 | Phosphorylation | TIGTRYVELFPSTPEEVSRARTRGR |
| XP_008656008.1 | S527 | Phosphorylation | NKEPGTISLTSGSPVKAARGDSASG |
| XP_003539633.1 | S18 | Phosphorylation | CVAAKDKTIQSASPSEILHRNIRHS |
| XP_006342746.1 | S1076 | Phosphorylation | SSPMRMLNMSKASPARMESSGKDDA |
| BAF02010.1 | S18 | Phosphorylation | GLYRPTQSVSPRSPLSPTRISPELL |
| BAF02010.1 | S21 | Phosphorylation | RPTQSVSPRSPLSPTRISPELLSPE |
| XP_003539789.1 | S146 | Phosphorylation | QQQLPESMIELISPDKPLPEKGKLL |
| NP_001142546.1 | T100 | Phosphorylation | PAAEPSSVSEPSTPSKVRFTAYYGG |
| XP_008664645.1 | T77 | Phosphorylation | HVGKNVKKCTSTTPQALEAKEKCKK |
| DAA39943.1 | S14 | Phosphorylation | WSNSSSKSSVAASPADLRSAMLLSD |
| AFX67013.1 | S267 | Phosphorylation | EIEEDIRRPKYFSPSEAVEYGIIDM |
| AFW73850.1 | S469 | Phosphorylation | NMDFAEKDSRVVSSSIPSRGVSPRR |
| AFW73850.1 | S478 | Phosphorylation | RVVSSSIPSRGVSPRRRLASDGVDT |
| XP_006584319.1 | S452 | Phosphorylation | PPPIYSPPPPVNSPPPPVSSPPPPV |
| XP_006584319.1 | S459 | Phosphorylation | PPPVNSPPPPVSSPPPPVYSPPPSP |
| XP_006584319.1 | S518 | Phosphorylation | PPPVNSPPPPVSSPPPPVYSPPPPL |
| XP_006366632.1 | S111 | Phosphorylation | TPTDKKSITDYGSPEEFLSKVDYLL |
| NP_001183870.1 | S94 | Phosphorylation | PERATKGSIRLRSEAAAGGSRKSDG |
| DAA50185.1 | T27 | Phosphorylation | FARAARLGSRAATPPPRTGIENSPR |
| NP_001132417.1 | S108 | Phosphorylation | RQQQNSRIDAVASPDRECPGRGNNN |
| NP_177885.1 | S72 | Phosphorylation | KNGATKKVAKGKSPRKTTPKKCATK |
| NP_177885.1 | T77 | Phosphorylation | KKVAKGKSPRKTTPKKCATKNGIVA |
| NP_001144889.1 | Y50 | Phosphorylation | ARGAKAKAVASRYLTPPSKPTSISS |
| NP_001144889.1 | T52 | Phosphorylation | GAKAKAVASRYLTPPSKPTSISSSS |
| XP_008666811.1 | T71 | Phosphorylation | EKQSKLSAPRPGTPWTAAAGRDTGW |
| XP_006367412.1 | S110 | Phosphorylation | VFFKYEKRIRMRSPPEKVFEYFASF |
| NP_001152094.1 | S23 | Phosphorylation | FAPAVKIPRRSSSNSEGSGGGAGNK |
| ACN36545.1 | Y5 | Phosphorylation | ********MDPIYKPREEKRKEREE |
| NP_187184.2 | S339 | Phosphorylation | RTVNLGKKSASISPIRNTGKRSPKL |
| XP_006600846.1 | S346 | Phosphorylation | QEEFDDAFNMMLSPSKEESRPVKSQ |
| XP_008680969.1 | S37 | Phosphorylation | SNAGASYTLLASSPPSSVRNDGCSP |
| XP_006347644.1 | S78 | Phosphorylation | HGSQLPKTSEVNSPIKNEKVDRTPT |
| AAC05343.1 | S595 | Phosphorylation | SAKGSMNSDRSFSSSKDIGGKRFKP |
| XP_008656275.1 | S239 | Phosphorylation | RTGAPTVIRHGSSPSPSSTPLSPTQ |
| XP_008656275.1 | S241 | Phosphorylation | GAPTVIRHGSSPSPSSTPLSPTQQL |
| NP_001105597.1 | S36 | Phosphorylation | TAVTIALIAGIPSPSDPTPRLRRGV |
| NP_001105597.1 | T41 | Phosphorylation | ALIAGIPSPSDPTPRLRRGVLLRRL |
| XP_006352274.1 | T131 | Phosphorylation | SPLSSTPSTGSITPGPKRAKGRPPG |
