# Supplementary figures and images for: In-depth assembly of organ and development dissected Picrorhiza kurroa proteome map using mass spectrometry
Source: BMC Plant Biol. 2021 Dec 22;21:604. doi: 10.1186/s12870-021-03394-8 (PMC8693493; doi:10.1186/s12870-021-03394-8)

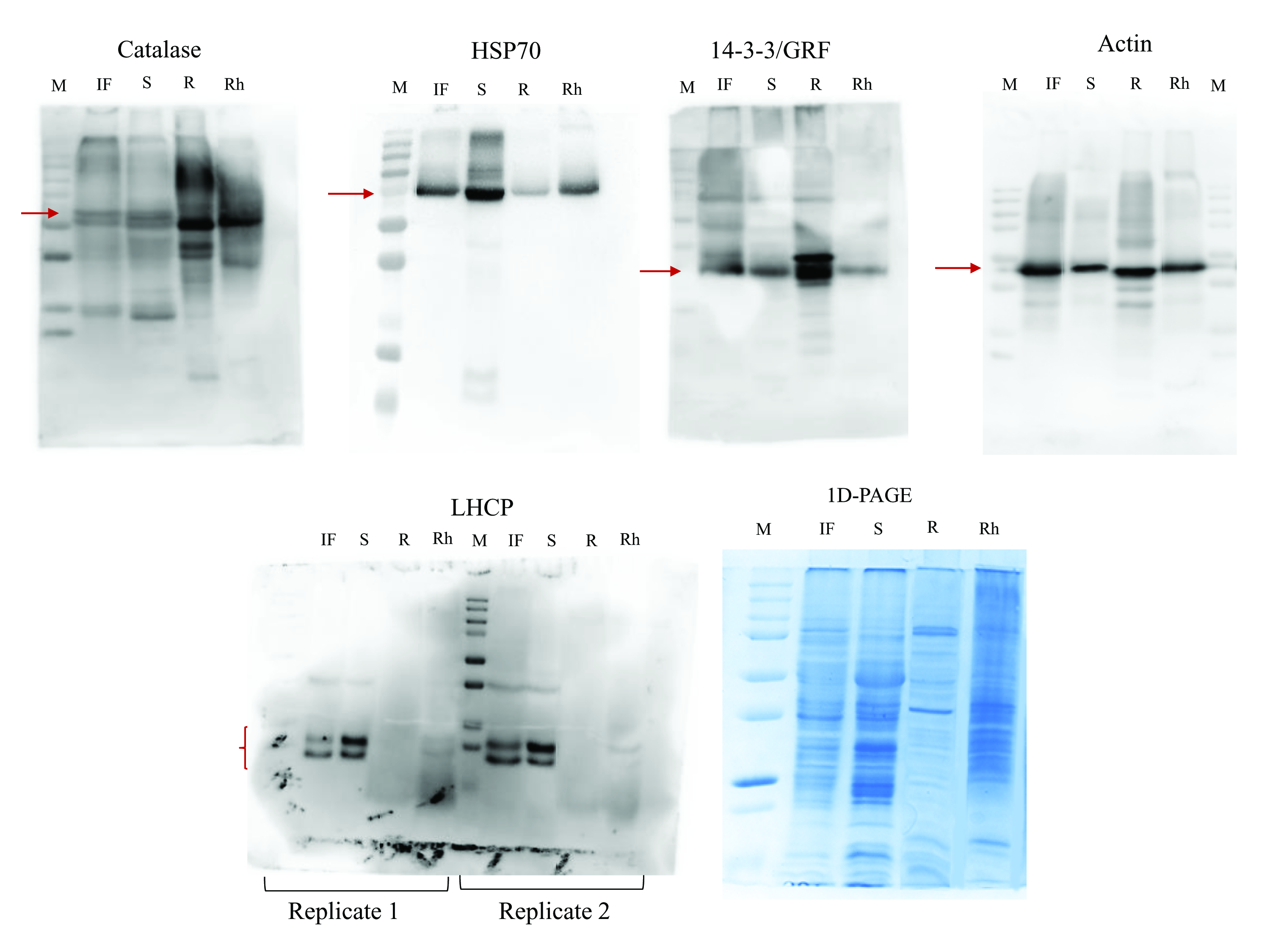

Supplement: Supplementary file 7 — Additional file 7: Fig. S1. Western blots and gel images in raw form for all the organ samples. The abbreviations used are as follows: IF, Inflorescence; S, Shoot; R, Root; Rh, Rhizome; M, Marker lane (10–250 kDa). [file 12870_2021_3394_MOESM7_ESM.tif]

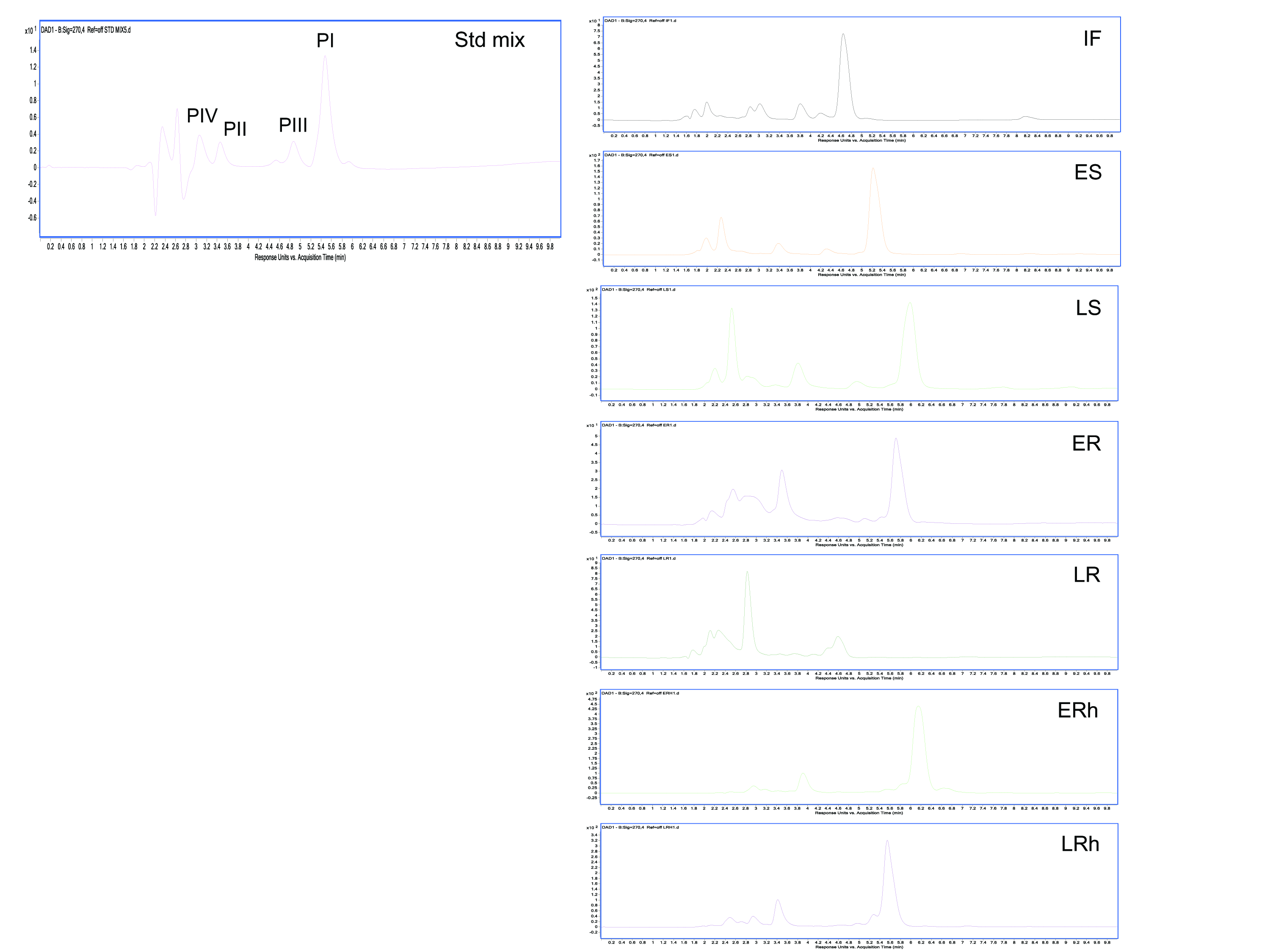

Supplement: Supplementary file 8 — Additional file 8: Fig. S2. UHPLC chromatograms of picrosides extracted in 70% methanol at 270 nm. The abbreviations used are: IF, Inflorescence; ES, Early shoot; LS, Late shoot; ER, Early root; LR, Late root; ERh, Early rhizome; LRh, Late rhizome; PI, Picroside I; PII, Picroside II; PIII, Picroside III; PIV, Picroside IV. [file 12870_2021_3394_MOESM8_ESM.tif]
